# Description of the Behavior of a Polymer Hybrid—A Mathematical and Numerical Review

**DOI:** 10.3390/ma19194054

**Published:** 2026-09-22

**Authors:** Betel Chinasho, Piotr Żach

**Affiliations:** 1 Division of Mechanical Engineering, College of Engineering, Wolaita Sodo University, Wolaita Sodo 109-2244, Ethiopia; 2 Faculty of Automotive and Construction Machinery, Warsaw University of Technology, Narbutta 84, 02-524 Warsaw, Poland; piotr.zach@pw.edu.pl

**Keywords:** hybrid materials, polymer, mathematical description, design, structure and morphology, mechanical behavior, numerical identification and modeling

## Abstract

Polymer hybrid materials have attracted considerable interest because combining different reinforcing constituents can provide improved mechanical performance, durability, lightweight characteristics, and structural efficiency. However, designing their behaviour remains challenging due to heterogeneous microstructures, constituent interactions, interfacial behaviour, reinforcement architecture, and manufacturing conditions. This review critically examines analytical, micromechanical, numerical, and reliability-based modelling approaches for polymer hybrid materials, covering their classification, mechanical characteristics, and predictive frameworks. Their applications in predicting tensile, flexural, and impact behaviour, damage, failure, and material degradation are discussed. The role of experimental characterization in determining material properties, model calibration, and numerical validation is also considered. Machine learning is emerging as a complementary tool for surrogate modelling within multiscale frameworks. The reviewed studies demonstrate variations between theoretical, numerical, and experimental results due to differences in material systems, fibre architecture, manufacturing and testing conditions, and modelling assumptions. This review highlights the need for reliable material datasets and unified modelling frameworks to improve predictive accuracy and support the design and optimization of polymer hybrid materials.

## 1. Introduction

Polymer composite materials have become one of the most important classes of engineering materials because of their superior specific strength, high stiffness-to-weight ratio, excellent corrosion resistance, and the ability to tailor their properties to meet specific engineering requirements [1,2].

Among the various classes of composite materials, polymer hybrid composites are characterized by the incorporation of two or more dissimilar reinforcing constituents within a common polymer matrix [3,4]. In these systems, the polymer matrix forms the continuous phase, while the different reinforcing constituents constitute distinct reinforcing phases. Hybridization refers to the deliberate combination of these reinforcing phases to exploit their complementary mechanical and functional characteristics while reducing the limitations associated with individual reinforcement systems. This approach provides opportunities to tailor stiffness, strength, fatigue resistance, fracture toughness, impact performance, and structural reliability for specific engineering applications [5,6].

The concept of hybridization has evolved considerably since the pioneering work on glass/carbon fibre hybrid composites demonstrated the so-called *positive hybridization effect*, in which the mechanical performance of hybrid composites exceeded that of composites reinforced with individual constituents [1]. Since then, numerous studies have investigated different combinations of synthetic fibres, natural fibres, particulate reinforcements, and nanofillers to improve the structural efficiency and multifunctional performance of polymer hybrid materials [3,4].

Nevertheless, the behaviour of hybrid polymer materials remains indeterminate and dependent on constituent selection, reinforcement architecture, fibre orientation, volume fraction, interfacial bonding, and manufacturing quality, resulting in significant variations in the reported mechanical properties and structural performance [6].

Although substantial progress has been achieved in the experimental characterization and computational analysis of polymer hybrid materials, accurately describing their mechanical behaviour remains a significant scientific challenge. The heterogeneous microstructure of hybrid materials gives rise to complex interactions between reinforcing constituents, polymer matrices, and interfacial regions operating across multiple length scales.

Consequently, the effective response of hybrid materials cannot be completely explained using simplified material theories developed for homogeneous or single-reinforcement composite systems [7,8]. These complexities have motivated the development of numerous analytical, micromechanical, constitutive, computational, and reliability-based models for predicting the effective behaviour of polymer hybrid materials.

Despite the availability of numerous mathematical approaches, no universally accepted modelling framework has yet been established. Existing mathematical descriptions differ considerably with respect to their underlying assumptions, constituent idealizations, treatment of interfacial interactions, and representation of damage evolution. As a result, discrepancies frequently arise between analytical predictions, numerical simulations, and experimental observations, thereby limiting the predictive capability and general applicability of many existing models [2,6]. These inconsistencies demonstrate the necessity of critically evaluating the current state of mathematical modelling and identifying the challenges that must be addressed to improve predictive accuracy.

Therefore, this paper presents a critical review of the mathematical description of the behaviour of polymer hybrid materials. The review first discusses the fundamental characteristics governing hybrid polymer materials before critically examining the principal analytical, micromechanical, constitutive, computational, and reliability-based modelling approaches reported in the literature. Particular emphasis is placed on the identification of material parameters, the assumptions and limitations of existing mathematical models, and the discrepancies between theoretical predictions and experimentally observed behaviour. Finally, current research gaps and future directions are highlighted to facilitate the development of more robust and unified mathematical frameworks for the analysis and design of polymer hybrid materials.

Figure 1 presents the schematic representation of the principal architectures of polymer hybrid materials. Specifically, Figure 1a illustrates laminate and multilayered laminate configurations, Figure 1b depicts hybrid composites reinforced with short fibres, and Figure 1c shows hybrid composites reinforced with long fibres. These representative architectures provide the fundamental basis for understanding the mechanical behaviour and subsequent mathematical description of polymer hybrid materials discussed throughout this review [3,6].

Therefore, this paper presents a critical review of the mathematical description of the behaviour of polymer hybrid materials. Section 1 introduces the definition and classification of polymer hybrid materials according to reinforcement source, reinforcement form, and structural configuration. Section 2 reviews their principal mechanical characteristics, with emphasis on tensile, flexural, and impact behaviour. Section 3 discusses statistical modelling and the calculation of deviations for describing material variability and degradation. Section 4 examines micromechanical modelling approaches for predicting the effective properties of hybrid materials. Section 5 addresses continuum-scale modelling and constitutive descriptions, while Section 6 discusses continuum-scale modelling of polymer composites. Section 7 focuses on viscoelastic and viscoplastic behaviour. Section 8 reviews advanced fracture and damage modelling approaches. Section 9 discusses numerical modelling approaches for polymer hybrid materials. Section 10 examines machine-learning-based approaches and their potential for surrogate and multiscale modelling. Section 11 identifies future research directions, and Section 12 summarizes the principal conclusions of the review.

### 1.1. Definition and Classification

Polymer hybrid materials represent a distinct class of composite materials in which two or more reinforcing constituents are incorporated within a common polymer matrix to achieve property combinations that cannot be attained using a single reinforcement system alone [2,3]. The hybridization concept exploits the complementary characteristics of different reinforcing phases to improve the overall mechanical response, durability, and structural efficiency of polymer composites while overcoming the limitations associated with individual constituent materials [1,4].

Consequently, polymer hybrid materials have become increasingly important in the development of lightweight structural systems requiring optimized combinations of stiffness, strength, fracture toughness, fatigue resistance, and impact performance.

The classification of polymer hybrid materials provides a systematic framework for understanding the diversity of reinforcement architectures and constituent combinations employed in engineering applications. Various classification schemes have been proposed in the literature, primarily based on the source of reinforcement, constituent configuration, reinforcement geometry, and fibre orientation. Although these classification systems facilitate material selection and structural design, they also demonstrate that the mechanical behaviour of hybrid materials is strongly influenced by the arrangement and interaction of their constituent phases rather than by reinforcement type alone [6]. Therefore, an appropriate classification of polymer hybrid materials establishes the necessary foundation for interpreting their mechanical behaviour and for developing suitable mathematical descriptions.

Based on the available literature, polymer hybrid materials may be classified according to the origin of the reinforcing constituents, reinforcement architecture, and structural configuration. Each classification reflects different mechanisms of load transfer, constituent interaction, and damage evolution, which subsequently influence the analytical, micromechanical, and computational models used to describe material behaviour. The principal classification systems adopted in the literature are discussed in the following subsections.

#### 1.1.1. Classification Based on Reinforcement Sources

One of the most widely adopted approaches for classifying polymer hybrid materials is according to the origin of the reinforcing constituents. The source and characteristics of the reinforcement play a fundamental role in determining the mechanical, thermal, and physical behaviour of hybrid polymer materials. Consequently, the selection and combination of reinforcing phases directly influence the constitutive response, stiffness, strength, damage evolution, and overall structural performance of the composite system [3,6]. Since the effective behaviour of hybrid materials originates from the interaction between individual constituents, the reinforcement source also constitutes one of the primary parameters considered in analytical, micromechanical, and computational modelling approaches.

From a mathematical perspective, the classification of polymer hybrid materials according to reinforcement sources provides a convenient basis for describing constituent properties within multiscale modelling frameworks. Material parameters such as elastic modulus, Poisson’s ratio, density, thermal expansion coefficient, and interfacial characteristics are generally assigned according to the individual reinforcing constituents before homogenization procedures are employed to estimate the effective behaviour of the hybrid system. Consequently, accurate identification of constituent materials represents an essential prerequisite for reliable mathematical descriptions and numerical simulations of polymer hybrid materials.

Based on the origin of the reinforcing constituents, polymer hybrid materials can be broadly classified into three categories: synthetic–synthetic, natural–synthetic, and natural–natural systems.

Synthetic–synthetic hybrids combine two or more dissimilar synthetic reinforcements within a common polymer matrix, whereas natural–synthetic hybrids integrate natural and synthetic reinforcing constituents.

Natural–natural hybrids, in contrast, incorporate two or more distinct natural reinforcements within the same matrix. This origin-based classification is distinct from classifications based on reinforcement geometry, morphology, or structural architecture; consequently, a hybrid material may belong to more than one classification category.

For example, a flax–glass/epoxy composite is classified as a natural–synthetic hybrid according to reinforcement origin and as a fibre-reinforced polymer hybrid according to reinforcement form. Such distinctions are particularly relevant to mathematical modelling, as differences in constituent properties, material variability, interfacial characteristics, and failure mechanisms influence the assumptions, parameters, and modelling strategies required to describe the effective behaviour of hybrid systems. The principal reinforcement-source categories and their associated characteristics are discussed in the following subsections.

#### 1.1.2. Synthetic–Synthetic Fibre-Reinforced Polymer Hybrid Materials

Synthetic fibres are extensively employed in structural applications because of their superior material characteristics, which enable the development of lightweight structures with enhanced mechanical performance compared with conventional metallic materials. Polymer hybrid materials reinforced with two or more dissimilar synthetic fibres provide a practical and cost-effective approach for achieving improved structural efficiency while satisfying specific engineering requirements [5].

Among the various synthetic reinforcements, glass fibre is widely utilized because of its relatively low cost and favourable mechanical properties. To further enhance structural performance, glass fibres are commonly hybridized with high-performance reinforcements such as carbon or graphite fibres. These fibres are preferred because of their low density, high specific strength, and high specific modulus, enabling the resulting polymer hybrid material to achieve an improved stiffness-to-weight ratio and overall mechanical performance while maintaining economic feasibility [5].

The hybridization of different synthetic fibres has facilitated the development of polymer hybrid materials with improved tensile strength, flexural strength, impact resistance, and stiffness for a wide range of structural applications [9,10].

These improvements arise from the complementary mechanical characteristics of the reinforcing constituents, which enhance the overall structural response of the composite system. Representative synthetic–synthetic fibre-reinforced polymer hybrid materials and their reported mechanical characteristics are summarized in Table 1.

From the perspective of mathematical description, synthetic–synthetic polymer hybrid materials provide an important foundation for developing analytical and computational models because the mechanical properties of their constituent fibres are generally well characterized. Parameters such as elastic modulus, density, fibre volume fraction, and constituent arrangement can be incorporated into mathematical formulations to estimate the effective behaviour of the hybrid material.

However, the interaction between different synthetic fibres, the quality of the fibre–matrix interface, and variations introduced during manufacturing may significantly influence stress transfer, stiffness, and failure behaviour, resulting in discrepancies between theoretical predictions and experimentally measured responses.

The studies summarized in Table 1 demonstrate the continuous evolution of synthetic–synthetic polymer hybrid materials through improvements in flexural strength, impact resistance, stiffness-to-weight ratio, and tensile strength achieved using different reinforcement combinations. Nevertheless, the reported improvements are often dependent on constituent selection, reinforcement architecture, manufacturing processes, and testing conditions, making direct comparison among different hybrid systems difficult.

#### 1.1.3. Natural–Synthetic Fibre-Reinforced Polymer Hybrid Materials

Natural fibres have attracted considerable attention as reinforcing constituents in polymer hybrid materials because of their high specific strength, low density, renewability, high stiffness, and relatively low production cost. Despite these advantages, natural fibres also exhibit several limitations, including lower impact strength, higher moisture absorption, and reduced durability compared with synthetic fibres [19]. These morphological drawbacks have motivated the development of natural–synthetic fibre-reinforced polymer hybrid materials to improve the overall mechanical and physical performance of natural fibre composites.

Most studies involving natural–synthetic polymer hybrid materials have focused on enhancing the mechanical and thermal characteristics of natural-fibre-reinforced composites through hybridization with synthetic fibres. Recent investigations have demonstrated that combining natural and synthetic fibres provides an effective balance between cost and mechanical performance compared with individual reinforcement systems [24]. Consequently, hybrid natural–synthetic fibre-reinforced polymers exhibit improved stiffness, strength, impact resistance, and durability while maintaining the environmental and economic advantages associated with natural fibres.

Carbon and glass fibres are frequently employed as synthetic reinforcements because they possess higher stiffness, strength, and resistance to moisture than natural fibres. It has been widely reported that the incorporation of glass fibres into natural fibre composites reduces property variability and moisture sensitivity [25]. Since natural fibres are inherently hydrophilic owing to their porous structure, they readily absorb moisture, which may result in swelling and deterioration of the composite properties. Consequently, hybridization with synthetic fibres, together with appropriate chemical modification of the natural fibres, has been proposed to overcome these limitations [26].

The combination of hydrophilic natural fibres with hydrophobic synthetic fibres improves the mechanical performance of polymer hybrid materials by reducing the deterioration of mechanical properties associated with moisture absorption [27]. Consequently, hybridization produces composite materials with enhanced mechanical characteristics while simultaneously reducing manufacturing cost. The advantages offered by one reinforcing constituent compensate for the limitations of the other, enabling the development of hybrid materials with improved overall performance [28].

Most current research has therefore focused on reducing the proportion of synthetic fibres while maintaining or improving the mechanical behaviour of hybrid composites [29]. Such an approach contributes to the development of structural materials capable of partially replacing expensive and non-renewable synthetic reinforcements [30].

Various combinations of natural and synthetic fibres have been investigated for structural applications. The hybridization of synthetic Polyolefin–Aramid (FORTA) fibres with jute fibres has been reported to improve crack growth resistance compared with jute-fibre-reinforced composites [31]. Similarly, the hybridization of cotton and glass fibres enhances the elastic modulus, tensile modulus, and impact modulus of polymer hybrid materials, although a reduction in material toughness has also been reported [32]. Increasing the proportion of synthetic fibres generally decreases moisture absorption while improving the elastic behaviour of the composite.

Furthermore, the incorporation of nano-silica-treated polypropylene macro-synthetic fibres enhances frictional shear stress, energy absorption, and residual strength during the post-cracking stage [33].

Natural fibres such as flax have also been successfully hybridized with carbon fibres for commercial applications, including bicycle frames and tennis rackets, because of their excellent vibration damping capability [25]. Comparative studies on different stacking sequences have shown that flax–carbon–flax configurations exhibit superior mechanical performance and greater impact energy absorption than carbon–flax–carbon laminates [34]. Likewise, the incorporation of glass fibres into flax fibre composites improves the overall structural strength [25].

Investigations on glass fibre/sisal fibre hybrid composites have further demonstrated that synthetic fibre reinforcement enhances the rigidity of adhesive joints compared with purely natural fibre composites [35]. Tajdini et al. reported that materials containing steel fibres exhibit higher flexural performance, ductility, and toughness than those reinforced with polypropylene and jute fibres [36]. Hybridization also affects the thermal stability of polymer hybrid materials, and functionally graded hybrid composites have been reported to exhibit superior thermal stability compared with pure vinylester resin systems [37].

Representative natural–synthetic fibre-reinforced polymer hybrid materials together with their reported mechanical characteristics are summarized in Table 2. The available studies demonstrate that natural–synthetic fibre hybridization provides an effective strategy for simultaneously improving mechanical performance, environmental sustainability, and economic feasibility.

#### 1.1.4. Natural Fibre Reinforced Polymer Hybrid Materials

Natural-fibre-reinforced polymer hybrid materials have emerged as attractive engineering materials because of their low density, renewability, biodegradability, and favourable specific mechanical properties. However, unlike synthetic reinforcements, natural fibres exhibit considerable variability in their physical and mechanical characteristics owing to differences in cultivation environment, geographical origin, plant age, and fibre extraction processes. These factors significantly influence fibre morphology, strength, and dimensions, thereby affecting the overall behaviour of polymer hybrid materials [62].

In addition, the performance of natural fibre composites depends on several interacting parameters, including fibre selection and processing/preparation, interfacial adhesion, matrix characteristics, porosity, and fibre orientation. Consequently, hybridization between different natural fibres has become an effective strategy for improving the structural performance and reliability of natural-fibre-reinforced polymer hybrid materials [19]. Vegetable-fibre-reinforced composites (VFRCs) have demonstrated considerable potential for structural applications because of their favourable mechanical properties. Furthermore, hybridization of different cellulosic fibres offers improved flexibility, enhanced mechanical performance, environmental sustainability, and economic benefits compared with conventional single-fibre composites [4].

Several studies have demonstrated the effectiveness of natural–natural fibre hybridization in enhancing composite performance. Herrera-Franco and Valadez-González reported that continuous henequen-fibre-reinforced high-density polyethylene (HDPE) composites exhibited significantly improved flexural strength in both the longitudinal and transverse directions through appropriate matrix selection [63].

Similarly, Alavudeen et al. investigated woven banana–kenaf hybrid composites and observed that hybridization produced superior mechanical properties compared with composites reinforced using either banana or kenaf fibres individually [64]. Senthil Kumar et al. further examined banana–coconut hybrid composites and showed that fibre stacking sequence significantly influenced flexural behaviour, with coconut sheath layers providing higher flexural strength than pure banana or coconut fibre composites [65].

Recent developments have also highlighted the increasing use of flax fibres as reinforcement in polymer composites because of their wide availability, low cost, and favourable mechanical characteristics for structural applications [66]. In addition, studies on natural fibre laminates have demonstrated that stacking sequence and fibre architecture strongly influence the viscoelastic response of composite materials [67,68].

Alternative natural reinforcements and fibre modification techniques have further expanded the application of natural fibre hybrid materials. Basalt fibres have received increasing attention as environmentally friendly alternatives to conventional glass fibres because of their excellent chemical resistance and favourable environmental characteristics [69]. Likewise, chemical surface modification methods, including mercerization, acetylation, benzoylation, peroxide treatment, and coupling-agent treatment, have been widely employed to improve fibre surface characteristics and strengthen fibre–matrix interfacial bonding [70].

Recent investigations have also explored advanced natural fibre composite systems, including sound-absorbing composite structures and nanomaterial-reinforced polymer composites, to improve multifunctional performance [71]. Furthermore, the incorporation of nanomaterials has been shown to improve tensile strength, fracture toughness, wear resistance, and overall mechanical performance through enhanced stress transfer between fibres and the surrounding polymer matrix [72].

Representative natural–natural fibre-reinforced polymer hybrid materials are summarised in Table 3. The reported studies demonstrate that hybrid combinations such as banana–sisal, banana–kenaf, banana–coconut, kenaf–bamboo–coir, sisal–cotton, jute–sisal, jute–gongura, banana–jute, and basalt–jute generally improve fibre–matrix interaction, mechanical strength, stiffness, durability, and thermal stability compared with corresponding single-fibre composites.

The mathematical description of polymer hybrid materials varies mainly in model assumptions, parameterization, and uncertainty rather than in the modelling frameworks themselves. Synthetic–synthetic systems generally involve well-characterized constituents, whereas natural–synthetic and natural–natural systems require greater consideration of constituent variability, morphology, environmental effects, and interfacial behaviour. Consequently, model parameterization and calibration become increasingly important for reliable prediction across these hybrid systems. Table 4 summarizes the principal differences.

### 1.2. Based on Fibre Reinforcement

The classification of polymer hybrid materials based on the type of reinforcement is an important approach for understanding the wide range of hybrid composite systems developed for engineering applications. The type, geometry, and distribution of the reinforcing phase significantly influence the mechanical, thermal, and functional performance of polymer hybrid materials. By selecting suitable reinforcement forms, hybrid materials can be tailored to achieve specific structural requirements, improved durability, lightweight characteristics, and multifunctional performance. Based on the reinforcement morphology, polymer hybrid materials can generally be classified into particle-reinforced, fibre-reinforced, and structural hybrid materials.

#### 1.2.1. Particle-Reinforced Polymer Hybrid Materials

Particle-reinforced polymer hybrid materials are produced by incorporating particles of different sizes and morphologies into a polymer matrix to enhance the overall mechanical and structural performance. The reinforcing particles may vary from fine dispersions to coarse particulates and may possess regular or irregular geometries depending on the intended application [82].

Depending on the strengthening mechanism, these materials are commonly categorized as large-particle reinforced or dispersion-strengthened polymer composites. The incorporation of particulate reinforcements improves stiffness, wear resistance, dimensional stability, and load transfer between the matrix and reinforcement. Concrete represents one of the classical examples of a particle-reinforced composite, where the chemical interaction between cement and aggregates provides the desired structural performance [83].

#### 1.2.2. Fibre-Reinforced Polymer Hybrid Materials

Fibre-reinforced polymer (FRP) hybrid materials consist of polymer matrices reinforced with one or more types of fibres to obtain superior mechanical performance. Common reinforcing fibres include glass, carbon, aramid, basalt, flax, hemp, jute, sisal, bamboo, and coir fibres, while thermosetting matrices such as epoxy, vinyl ester, polyester, and phenolic resins are widely employed because of their excellent bonding characteristics and structural integrity [31].

Fibre-reinforced polymer hybrid materials have found widespread applications in aerospace, automotive, marine, civil infrastructure, and energy sectors because of their high specific strength, stiffness, corrosion resistance, and lightweight nature [31,84,85]. Continuous fibre-reinforced polymer hybrids generally provide superior tensile strength, stiffness, and vibration damping characteristics compared with discontinuous fibre systems. In contrast, short or randomly oriented fibres are often adopted to reduce manufacturing costs and facilitate large-scale industrial processing while maintaining acceptable mechanical performance [83,86].

#### 1.2.3. Structural Polymer Hybrid Materials

The structural performance of polymer hybrid materials depends not only on the properties of their constituent materials but also on the geometric arrangement of the reinforcing elements and laminate architecture [30]. Structural polymer hybrid materials are generally classified into laminated composites and sandwich composites.

Laminated polymer hybrid materials comprise multiple reinforcement layers embedded within a polymer matrix, manufactured using glass, carbon, natural fibres, metals, or woven fabrics to achieve enhanced stiffness, strength, and fatigue resistance at low weight. Stacking sequence, fibre orientation, and layer configuration critically govern structural behaviour.

Sandwich polymer hybrid materials consist of thin, high-strength face sheets bonded to a lightweight foam, honeycomb, or polymeric core. This configuration provides excellent bending stiffness, energy absorption, and vibration damping, making them attractive for aerospace, marine, transportation, and civil engineering applications [87].

### 1.3. Applications of Polymer Hybrid Materials

Polymer hybrid materials have attracted considerable interest for engineering applications because their properties can be tailored through the combination of different reinforcing constituents within a polymer matrix. The resulting balance of strength, stiffness, lightweight characteristics, durability, and functional performance makes these materials suitable for applications across aerospace, marine, military, sports, construction, and consumer-product sectors [88].

The major application areas of polymer hybrid materials include the following:

- **Aerospace applications:** Polymer hybrid materials are employed in components such as wing structures, interior panels, seats, tail components, and other body structures, where lightweight construction and adequate mechanical performance are essential.
- **Military and defence applications:** Their applications include protective systems such as bullet-resistant jackets, military vehicles, and components of warships, where weight reduction, structural integrity, and damage resistance are important considerations.
- **Electrical and electronic applications:** Polymer-based hybrid materials are used in products such as laptops, coolers, watches, and other electronic components, where combinations of mechanical, dimensional, and functional properties are beneficial.
- **Construction applications:** Hybrid composite materials can be used in structural components where reduced weight, adequate mechanical performance, and improved durability are required.
- **Packaging applications:** Polymer hybrid systems also have potential in food-packaging applications, where material selection can be tailored to provide an appropriate combination of mechanical and functional properties.

Despite their broad application potential, the suitability of a particular hybrid material depends strongly on the reinforcement combination, matrix system, interfacial bonding, manufacturing process, and service environment. Therefore, application-specific optimization remains essential for achieving an appropriate balance among mechanical performance, durability, weight, cost, and manufacturability.

## 2. Mechanical Characteristics of Polymer Hybrid Materials

The mechanical characteristics of polymer hybrid materials have attracted considerable research attention because they govern the suitability of these materials for load-bearing engineering applications. Hybridization combines two or more reinforcing constituents within a single polymer matrix to achieve mechanical properties that cannot be attained using individual reinforcements alone.

This synergistic approach enables improvements in tensile strength, stiffness, toughness, impact resistance, fatigue performance, and damage tolerance, etc., making polymer hybrid materials attractive for aerospace, automotive, marine, civil infrastructure, and other advanced engineering applications [3,4,5]. Nevertheless, their mechanical behaviour is influenced by complex interactions among fibre properties, fibre architecture, matrix characteristics, interfacial bonding, manufacturing techniques, and environmental conditions. Consequently, understanding these interactions is essential for both material property design and the development of reliable constitutive and numerical models capable of accurately predicting the mechanical response of polymer hybrid materials.

Although numerous experimental investigations have demonstrated the benefits of fibre hybridization, direct comparison among reported studies remains challenging because of differences in constituent materials, fibre volume fractions, stacking sequences, manufacturing techniques, and testing conditions.

Furthermore, most studies focus primarily on experimental characterization, whereas comparatively fewer investigations establish predictive mathematical models capable of describing the observed mechanical behaviour. Therefore, this section critically reviews the available literature on the principal mechanical properties of polymer hybrid materials while highlighting the existing challenges and opportunities for analytical modelling, constitutive formulation, and numerical simulation.

### 2.1. Tensile Properties

Tensile properties are among the most important mechanical characteristics used to evaluate the structural performance of polymer hybrid materials because they directly reflect the material’s load-carrying capability and resistance to deformation under axial loading. The tensile response is governed by several interacting factors, including fibre type, fibre orientation, fibre volume fraction, interfacial adhesion, matrix characteristics, stacking sequence, and manufacturing quality. These parameters collectively determine the efficiency of stress transfer between the reinforcing fibres and the surrounding polymer matrix, thereby influencing both tensile stiffness and ultimate tensile strength.

Several experimental studies have demonstrated that fibre hybridization effectively enhances the tensile behaviour of polymer hybrid materials. Indra Reddy et al. reported that epoxy composites reinforced with jute, pineapple leaf fibre, and glass fibre exhibited an increase in tensile modulus with increasing fibre volume fraction, indicating improved stress transfer between the reinforcing fibres and the polymer matrix [89]. Similarly, Aslan et al. compared waste sisal/glass and sisal/carbon-fibre-reinforced polypropylene hybrid composites and observed that the incorporation of carbon fibres significantly increased the tensile modulus because of their higher stiffness [90]. Their study further demonstrated that increasing the carbon-to-glass fibre ratio progressively enhanced the tensile performance of the hybrid composite.

Hybridization has also been shown to improve the tensile characteristics of short coir/glass-fibre-reinforced polymer composites fabricated using the hand lay-up technique. Increasing the coir fibre content enhanced the tensile strength, while fibre length was identified as another important parameter affecting tensile behaviour [91]. Likewise, Thakur et al. demonstrated that incorporating carbon nanotubes into bamboo-fibre-reinforced polymer composites increased tensile strength as the nanotube content increased from 1% to 3%, highlighting the effectiveness of nanoscale reinforcements in improving stress transfer within hybrid composite systems [92].

Further improvements in tensile performance have been reported for banana–jute–glass fibre hybrid composites, where hybridization resulted in superior overall mechanical properties compared with individual fibre-reinforced composites [93]. Fibre orientation has also been identified as a critical factor governing tensile behaviour. Kaliappan et al. observed that neem–abaca fibre hybrid laminates with fibres oriented at $45^{\circ }$ exhibited superior tensile performance compared with horizontal and vertical fibre arrangements because the inclined orientation promoted more effective stress redistribution throughout the laminate [94].

Similarly, Yahaya et al. demonstrated that woven kenaf–aramid (Kevlar) hybrid laminates exhibited improved tensile properties when the Kevlar layers were placed on the outer surfaces, emphasizing the importance of stacking sequence and laminate architecture in hybrid composite design [41]. Furthermore, Veerasimman et al. reported that introducing red mud as a secondary particulate reinforcement into banana-fibre-reinforced polyester composites further increased tensile strength, with the improvement depending on both the filler particle size and reinforcement content [95].

Despite the consistent improvements reported in tensile performance, the existing literature also reveals important limitations. Most investigations focus on specific material combinations and manufacturing conditions, making it difficult to establish generalized relationships between hybrid architecture and tensile response. In addition, experimental observations are often presented without corresponding predictive mathematical models capable of describing stress transfer, interfacial debonding, damage evolution, and progressive failure.

### 2.2. Flexural Properties

Flexural properties are important indicators of the structural performance of polymer hybrid materials because they describe the material’s ability to resist bending loads while maintaining stiffness and structural integrity. The flexural behaviour of hybrid composites is governed by several interacting parameters, including fibre type, fibre orientation, stacking sequence, fibre length, fibre volume fraction, interfacial bonding, and the mechanical properties of the polymer matrix. Since many engineering components experience combined tensile and compressive stresses during service, flexural performance provides valuable insight into the load-bearing capability and failure mechanisms of polymer hybrid materials.

Several experimental investigations have demonstrated that hybridization can substantially improve the flexural behaviour of polymer hybrid materials. Dong et al. experimentally investigated the flexural response of S-glass/carbon-fibre-reinforced hybrid composites by varying the amount of glass fibres positioned on the outer carbon fibre layers [96]. Their results showed that increasing the glass fibre content reduced the flexural modulus, indicating that fibre distribution and constituent stiffness significantly influence the bending response of hybrid laminates.

The influence of stacking sequence on the flexural performance of basalt–carbon fibre laminated composites has also been investigated. Experimental three-point bending tests conducted according to ASTM D970-07 demonstrated that laminates containing carbon fibre layers exhibited higher flexural strength and modulus than those reinforced primarily with basalt fibres because of the superior stiffness of carbon reinforcement [97]. Similarly, Masoumi et al. investigated the effect of intralayer hybridization between basalt and carbon fibres and reported that hybrid laminates exhibited nearly identical flexural behaviour in both longitudinal and transverse directions, suggesting that an appropriate fibre arrangement can effectively reduce directional dependence in bending performance [98].

Nanomaterial reinforcement has also been explored to improve the flexural characteristics of polymer hybrid materials. Hybrid composites reinforced with micro-scale carbon fibres and nano-scale graphene oxide demonstrated an increase in Young’s modulus with increasing graphene oxide content. Although the addition of graphene oxide slightly reduced tensile strength, a loading of 0.5 wt.% produced approximately a 10% improvement in elastic modulus, illustrating the potential of multiscale hybrid reinforcement for enhancing stiffness [99].

Natural fibre hybridization has likewise demonstrated favourable effects on flexural behaviour. Akash et al. investigated sisal/coir-fibre-reinforced epoxy hybrid composites containing *Samanea saman* pod (SSP) pulp and reported that both flexural and tensile strengths increased with SSP content up to 10 wt.% [100]. However, further increases in filler concentration resulted in deterioration of the mechanical properties, indicating the existence of an optimum filler loading for achieving maximum reinforcement efficiency. Likewise, experimental investigations on woven jute and oil palm empty fruit bunch fibre-reinforced epoxy hybrid composites demonstrated that sandwich hybrid laminates exhibited considerably higher flexural strength than composites reinforced solely with oil palm fibres, confirming the beneficial effect of fibre hybridization on bending performance [101].

### 2.3. Impact Properties

Impact properties are critical for evaluating the ability of polymer hybrid materials to withstand sudden loading, absorb impact energy, and resist damage initiation and propagation under dynamic conditions. The impact behaviour of polymer hybrid materials is governed by several factors, including fibre type, fibre architecture, stacking sequence, matrix properties, interfacial bonding, and manufacturing quality. Owing to the synergistic interaction between different reinforcing constituents, hybridization has become an effective strategy for improving the damage tolerance and energy absorption capability of polymer composite systems.

Several experimental studies have demonstrated that fibre hybridization significantly enhances the impact performance of polymer hybrid materials. Hybrid epoxy composites reinforced with woven coir and E-glass fibres exhibited substantially higher impact strength than composites reinforced solely with natural fibres, confirming the beneficial contribution of glass fibres to impact resistance [91]. Similarly, Wang et al. investigated hybrid composites reinforced with carbon and polyimide fibres to evaluate the influence of fibre orientation on failure mechanisms, Charpy impact strength, and flexural behaviour [102]. The results showed that appropriate fibre orientation increased the Charpy impact strength by approximately 50%, demonstrating the important role of fibre architecture in improving impact resistance.

The incorporation of functional reinforcements has also been explored to enhance impact behaviour. Meo et al. investigated smart thermoplastic hybrid composites containing embedded shape memory alloy (SMA) wires subjected to low-velocity impact loading [103]. Their results demonstrated that embedding SMA wires significantly improved damage resistance and ductility compared with conventional composite laminates, indicating the potential of multifunctional hybrid systems for impact-resistant structures.

The influence of fibre hybridization on impact performance has also been examined for flax/carbon and flax/glass fibre hybrid composites modified with multi-walled carbon nanotubes. Compression-after-impact tests revealed that flax/glass hybrid composites exhibited superior impact resistance compared with flax/carbon systems, demonstrating that the selection of reinforcing fibres strongly influences post-impact mechanical behaviour [104]. Likewise, Ahmad Nadzri et al. reviewed low-velocity impact behaviour in kenaf/glass-fibre-reinforced hybrid laminates and concluded that fibre loading, fibre orientation, and surface treatment collectively govern impact performance [105]. Properly designed hybrid laminates were capable of withstanding impact energies of up to 12 J without catastrophic failure.

The stacking configuration of hybrid laminates also plays a significant role in determining impact behaviour. Pegoretti et al. demonstrated that both stacking sequence and constituent composition influence crack propagation during impact loading in E-glass/poly(vinyl alcohol) (PVA) woven hybrid composites [106]. In particular, intraply hybrid laminates exhibited greater resistance to impact-induced crack growth than interply laminates, while the incorporation of PVA fibres improved the specific impact energy of conventional E-glass laminates. Similarly, Venkata Reddy et al. reported that polyester hybrid composites reinforced with kapok, glass, and sisal fibres exhibited an increase in impact strength of approximately 34%, confirming the effectiveness of combining natural and synthetic fibres to improve impact energy absorption [107].

Further improvements have been reported for woven basalt–aramid hybrid composites. Wang et al. showed that interply hybrid laminates exhibited greater ductility, higher energy absorption, and lower peak impact loads than intraply laminates [108]. Post-impact damage analysis further revealed that interply laminates failed progressively through layer-by-layer delamination, whereas intraply laminates exhibited relatively brittle fracture, resulting in lower energy absorption capability.

Table 5 summarises representative studies on the mechanical characteristics of polymer hybrid materials. The reported investigations demonstrate that tensile, flexural, and impact properties can be significantly improved through appropriate selection of fibre combinations, stacking sequence, reinforcement architecture, and hybridization strategy.

However, the available studies also reveal considerable variability in mechanical performance arising from differences in constituent materials, manufacturing methods, and testing procedures. These observations highlight the need for unified mathematical models capable of accurately predicting the mechanical behaviour of polymer hybrid materials under different loading conditions and facilitating their efficient structural design.

## 3. Statistical Modelling and Calculation of Deviations

Isametova et al. developed a mathematical reliability model for polymer composite materials based on the statistical analysis of strength and degradation behaviour [109]. Their approach considers the random variation of stress and strength and evaluates the mean degradation, dispersion, standard deviation, and variation coefficient using a normal (Gaussian) distribution.

Figure 2 presents the test specimens investigated by Isametova et al., including the unfilled polymer composite and composites containing 20% and 30% short glass fibres [109]. These specimens were used to investigate the degradation behaviour of the polymer composite materials, which subsequently formed the basis for the statistical calculations described below.

For polypropylene-based polymeric materials, the yield point is considered a characteristic representing the ultimate strength of the material [110]. The random variation in stress and strength associated with structural elements manufactured from polymeric materials can be described using a normal distribution [111].

A normal, or Gaussian, distribution represents the probability distribution of a random variable. For the one-dimensional case, its probability density function can be written as [112]:

(1) $$f\left(x\right)=\frac{1}{\sigma \sqrt{2\pi }}\exp\left[-\frac{1}{2}{\left(\frac{x-\mu }{\sigma }\right)}^{2}\right]$$

where μ denotes the mathematical expectation (mean), which also corresponds to the median and mode of the distribution, while σ represents the standard deviation and $\sigma^{2}$ is the variance of the distribution.

1. Mathematical expectation of material degradation
  The mean degradation time of the material can be calculated by considering the degradation time associated with each interval and the corresponding number of sample failures:
  (2) $$L_{o}=\frac{\sum_{i=1}^{k}L_{i}m_{i}}{\sum_{i=1}^{k}m_{i}}$$
  where $L_{i}$ is the degradation time in the *i*-th interval, expressed in hours; $m_{i}$ is the number of sample failures in the *i*-th interval; and *k* represents the total number of intervals.
2. Dispersion of material degradation
  The dispersion of material degradation can be determined from the squared deviation of each degradation time from the mean degradation time:
  (3) $$D=\sigma^{2}=\frac{\sum_{i=1}^{k}{\left(L_{i}-L_{o}\right)}^{2}m_{i}}{\sum_{i=1}^{k}m_{i}}$$
3. Standard deviation or scattering of material degradation
  The standard deviation, representing the scattering of the degradation values, is obtained from the square root of the dispersion:
  (4) $$\sigma =\sqrt{D}=\sqrt{\frac{\sum_{i=1}^{k}{\left(L_{i}-L_{o}\right)}^{2}m_{i}}{\sum_{i=1}^{k}m_{i}}}$$
4. Variation coefficient
  The coefficient of variation, which represents the relative dispersion of the material degradation values, is defined as
  (5) $$V=\frac{\sigma }{L_{o}}$$

By processing the statistical data using Equations (12)–(15) and determining the numerical characteristics of degradation for the tested samples, an important characteristic of the material degradation process can be established. In particular, these calculations make it possible to identify the degradation stage at which the maximum number of sample failures occurs [113].

## 4. Analytical Models for Hybrid Composite Response

Tavares et al. [114] developed a series of analytical and computational models to investigate the tensile failure behaviour of hybrid polymer composites. Their study considered three levels of modelling complexity: a model for dry fibre bundles based on the statistical distribution of fibre strength, a progressive damage model based on fibre fragmentation, and a computational micromechanical model that accounts for the random distribution and stochastic strength of the fibres. These approaches were developed to investigate the mechanisms governing hybridization, failure sequence, and pseudo-ductile behaviour in hybrid composites.

The first analytical model proposed by Tavares et al. [114] considers dry bundles containing two different types of fibres. The model does not explicitly account for the polymer matrix and instead focuses on the influence of the statistical fibre-strength distributions and hybrid fibre volume fractions on the tensile failure behaviour of the fibre bundle. This simplified approach is useful for analysing the interaction between the strength distributions of the two fibre populations.

The model considers a bundle containing two types of fibres, denoted as fibre types 1 and 2, with a total number of fibres given by

(6) $$N_{t}=N_{t1}+N_{t2}.$$

The tensile strength of the individual fibres is assumed to follow a Weibull distribution [115]. The cumulative probability of fibre failure is expressed as [114]

(7) $$P\left(\sigma \right)=1-\exp\left[-\frac{L}{L_{0}}{\left(\frac{\sigma }{\sigma_{0}}\right)}^{m}\right],$$

where P(σ) is the cumulative probability of failure of a fibre with length *L* when subjected to a stress σ. The parameters $\sigma_{0}$ and *m* represent the Weibull scale and shape parameters, respectively, while $L_{0}$ denotes the characteristic reference length. A random number between zero and one can be generated to represent the cumulative failure probability of each fibre, from which the corresponding tensile strength of the individual fibre can be determined.

Because the model considers a hybrid composite containing two different fibre types, separate tensile-strength distributions are generated for each fibre population according to their respective fibre properties. The volume fractions of the two fibre types are related by

(8) $$V_{f1}+V_{f2}=1.$$

The model is based on the global load-sharing assumption, whereby the load released by a failed fibre is redistributed among the remaining fibres. Strain-controlled loading is considered, with the applied strain increased incrementally from zero by a prescribed strain increment Δε. Assuming linear elastic behaviour of the individual fibres, the stress in each fibre type is calculated as

(9) $$\sigma_{i}=E_{fi}\varepsilon ,$$

where $E_{fi}$ represents Young’s modulus of fibre type *i*, and ε is the applied strain.

When the tensile strength of an individual fibre is reached, that fibre is considered to have failed, and the number of broken fibres of the corresponding type is increased. The stress carried by the remaining fibres is then redistributed according to the global load-sharing assumption. For a bundle containing fibres with different radii, the average stress in the bundle can be expressed as

(10) $$\bar{\sigma }=\frac{\left(N_{t1}-N_{b1}\right)S_{f1}E_{f1}+\left(N_{t2}-N_{b2}\right)S_{f2}E_{f2}}{N_{t}\left(S_{f1}V_{f1}+S_{f2}V_{f2}\right)}\varepsilon ,$$

where $S_{fi}=\pi R_{fi}^{2}$ is the cross-sectional area of fibre type *i*, $R_{fi}$ is its radius, $N_{ti}$ is the initial number of fibres of type *i*, and $N_{bi}$ is the number of broken fibres of that type. The parameters $E_{fi}$ and $V_{fi}$ denote Young’s modulus and volume fraction of the corresponding fibre type, respectively.

For the subsequent analysis, fibre bundles containing 500 fibres with a gauge length of 75 mm are considered. The volume fraction of each fibre type is varied by changing the number of fibres belonging to each type, while maintaining the total number of fibres in the bundle at 500. The fibre properties used as input parameters for the analytical models are summarized in Table 6.

The parameters listed in Table 6 provide the material and statistical characteristics required for the analytical description of the fibre bundle. In particular, Young’s modulus determines the elastic response of each fibre type, whereas the Weibull parameters describe the statistical distribution of fibre tensile strength. Consequently, variations in the fibre properties and their relative volume fractions can be incorporated into the model to evaluate their influence on the tensile response and failure behaviour of the hybrid fibre bundle.

## 5. Microscale Modelling and Micromechanics of Polymer Composites

### 5.1. Microscale Modelling of Polymer Composites

Microscale modelling represents an intermediate level between the constituent and mesoscale scales by explicitly resolving a representative portion of the composite microstructure containing multiple reinforcements within the polymer matrix. Typically spanning $10^{-4}$ mm to $10^{-1}$ mm, it accounts for the geometric arrangement, spatial distribution, and orientation of reinforcements while remaining sufficiently small for continuum simulations. This scale enables the estimation of effective composite properties and provides essential input for hierarchical homogenization and multiscale simulations.

A central concept in microscale modelling is the representative volume element (RVE), defined as the smallest material volume that statistically captures microstructural heterogeneities while providing an averaged response representative of the macroscopic behaviour. For fibre-reinforced polymer composites, RVEs commonly consist of fibres embedded in a polymer matrix with either periodic or statistically equivalent distributions, as shown in Figure 3a,b.

Balasubramani et al. [120] investigated the selection of 3D RVE size and shape for unidirectional fibre-reinforced polymer composites, considering homogenized stiffness and local-field statistics related to strength and damage initiation. Using Monte Carlo simulations of random fibre arrangements, they evaluated the convergence of microstructural descriptors and predicted properties and compared random 3D RVEs with periodic unit-cell models. Wang et al. [121] developed an RVE-based multiscale framework for thermo-oxidative ageing in particle-filled polymer composites by incorporating ageing-dependent matrix and interface properties. A random particle-packing algorithm was used to construct the RVE, with uniform displacement boundary conditions applied for tensile loading. Trias et al. [122] introduced the statistical representative volume element (SRVE) for carbon-fibre-reinforced polymers to overcome limitations associated with conventional periodic RVEs. They proposed that an SRVE should satisfy both mechanical convergence criteria and point-pattern statistics to represent random fibre arrangements and related microscale damage mechanisms.

Although microscale modelling captures important microstructural mechanisms, it is generally integrated into hierarchical or multiscale frameworks, where microscale information is transferred to mesoscale and macroscale models [123]. Recent developments include image-based geometric models [124], physics-informed surrogate models [125], and ML-assisted homogenization [126], which aim to improve predictive accuracy while reducing computational cost.

### 5.2. Micromechanics-Based Models

Consider a two-phase composite laminate consisting of unidirectional fibres embedded in a matrix, as investigated in [127]. The fibres are aligned along direction 1 and are assumed to be uniformly distributed, such that the spacing between adjacent fibres is identical. A perfect interface is assumed between the fibres and the matrix.

Since the composite is reinforced unidirectionally within a layer, it is generally considered transversely isotropic, meaning that the material properties in directions 2 and 3 are identical. The composite is subjected to a homogeneous stress or strain state, and the overall effective stiffness of the composite can be expressed as

(11) σ=[C]{ϵ}

or

(12) {ϵ}=[S]{σ}

where

(13) $$\left\{\sigma \right\}={\left\{\sigma_{11},\sigma_{22},\sigma_{33},\sigma_{23},\sigma_{13},\sigma_{12}\right\}}^{T},$$

(14) $$\left\{\epsilon \right\}={\left\{\epsilon_{11},\epsilon_{22},\epsilon_{33},2\epsilon_{23},2\epsilon_{13},2\epsilon_{12}\right\}}^{T}$$

and [S] and [C] represent the compliance and stiffness matrices of the material, respectively. For a transversely isotropic material, the isotropy in the plane normal to the fibre direction leads to the following relationships among the engineering constants:

(15) $$E_{22}=E_{33},\;G_{13}=G_{12},\;\nu_{13}=\nu_{12}$$

The compliance matrix for a transversely isotropic material can be written as

(16) $$\left[S\right]=\left[\begin{array}{cccccc} \frac{1}{E_{11}} & -\frac{\nu_{12}}{E_{11}} & -\frac{\nu_{12}}{E_{11}} & 0 & 0 & 0\\ -\frac{\nu_{12}}{E_{11}} & \frac{1}{E_{22}} & -\frac{\nu_{23}}{E_{22}} & 0 & 0 & 0\\ -\frac{\nu_{12}}{E_{11}} & -\frac{\nu_{23}}{E_{22}} & \frac{1}{E_{22}} & 0 & 0 & 0\\ 0 & 0 & 0 & \frac{1}{G_{23}} & 0 & 0\\ 0 & 0 & 0 & 0 & \frac{1}{G_{12}} & 0\\ 0 & 0 & 0 & 0 & 0 & \frac{1}{G_{12}} \end{array}\right]$$

where $E_{11}$ and $E_{22}$ denote the effective elastic moduli in directions 1 and 2, respectively, while $\nu_{12}$ and $\nu_{23}$ are the Poisson’s ratios and $G_{12}$ and $G_{23}$ are the effective shear moduli in the 1-2 and 2-3 planes, respectively. The stiffness matrix of the composite is determined through the evaluation of effective properties, including the elastic modulus, shear modulus, and Poisson’s ratio. In the present review, the Voigt and Reuss models, Hashin–Shtrikman model, Chamis model, and Halpin–Tsai model are considered.

These approaches require limited microstructural information, such as the fibre volume fraction, and provide direct expressions for the effective mechanical properties, which can subsequently be used to construct the stiffness matrix of the composite. Other approaches, including the Mori–Tanaka model, Self-Consistent model, and differential scheme-based model, directly estimate the composite stiffness matrix using continuum mechanics approaches. The following sections provide a detailed review of these models.

#### 5.2.1. Rule of Mixtures

The Rule of Mixtures (RoM) is one of the simplest approaches for estimating the effective elastic properties of composite materials. In general, two approaches are commonly considered: the Voigt model [128], which is associated with longitudinal loading, and the Reuss model for transverse loading [129]. Under longitudinal loading in the Voigt model, the strain in both the fibre and matrix is assumed to be identical to the strain in the effective composite medium. The effective elastic modulus under longitudinal loading is therefore given by

(17) $$E_{11}=E_{1f}V_{f}+E_{m}V_{m}=E_{1f}V_{f}+E_{m}\left(1-V_{f}\right)$$

where $E_{11}$ denotes the effective modulus along the longitudinal or fibre direction, $E_{1f}$ is the elastic modulus of the fibre, and $E_{m}$ is the elastic modulus of the matrix, which is assumed to be isotropic. $V_{f}$ and $V_{m}$ represent the fibre and matrix volume fractions, respectively. The modulus obtained from the RoM represents the upper bound of the effective elastic modulus [130]. In contrast, the Reuss model assumes an iso-stress condition under transverse loading, where the stress carried by the fibre is transferred to the matrix and is equal to the stress in the effective representative volume element. For transverse loading, the effective modulus is expressed as

(18) $$\frac{1}{E_{22}}=\frac{V_{f}}{E_{22f}}+\frac{1-V_{f}}{E_{m}}$$

where $E_{22}$ is the transverse elastic modulus of the composite, $E_{22f}$ is the corresponding modulus of the fibre, and $E_{m}$ is the modulus of the matrix. This relationship is also referred to as the inverse rule of mixtures (iRoM). Other effective properties predicted using the RoM and iRoM include the Poisson’s ratio $\left(\nu_{12}\right)$, given by

(19) $$\nu_{12}=\nu_{12f}V_{f}+\nu_{m}V_{m}$$

The effective shear modulus $\left(G_{12}\right)$ under an iso-shear stress condition is

(20) $$\frac{1}{G_{12}}=\frac{V_{f}}{G_{12f}}+\frac{1-V_{f}}{G_{m}}$$

whereas the effective shear modulus $\left(G_{12}\right)$ under an iso-shear strain condition is

(21) $$G_{12}=G_{12f}V_{f}+G_{m}\left(1-V_{f}\right)$$

A more representative description of shear and bending behaviour can be obtained by assuming a parabolic shear stress distribution rather than an iso-shear stress condition.

#### 5.2.2. Effective Shear Modulus Based on Higher-Order Shear Stress Distribution

Consider a representative volume element comprising two different materials, with the fibre occupying the upper portion and the matrix occupying the lower portion. A higher-order transverse shear stress distribution is assumed across the long fibre and matrix layers, approximated by

(22) $$\tau \left(z\right)=Az^{2}+Bz+C$$

The shear stress at the upper surface $\left(z=h_{f}\right)$ and lower surface $\left(z=-h_{m}\right)$ is assumed to be zero. The remaining boundary condition is obtained from the shear force acting over the entire thickness,

(23) $$P=\int_{-h_{m}}^{h_{f}}\tau \left(z\right)dz$$

where *P* denotes the applied load in the shear-bending case. By solving for the three constants in Equation (10), the shear stress distribution becomes

(24) $$\tau \left(z\right)=\frac{3P}{h_{c}^{3}}\left[h_{f}h_{m}+\left(h_{f}-h_{m}\right)z-z^{2}\right]$$

where $h_{c}=h_{f}+h_{m}$. Using this higher-order parabolic shear stress distribution instead of the iso-shear stress assumption, the effective shear modulus $\left(G_{12}\right)$ is obtained as

(25) $$\frac{1}{G_{12}}=\frac{V_{f}^{2}\left(V_{f}+3V_{m}\right)}{G_{f}}+\frac{V_{m}^{2}\left(3V_{f}+V_{m}\right)}{G_{m}}$$

where $V_{f}$ and $V_{m}$ denote the fibre and matrix volume fractions, respectively. The longitudinal elastic modulus and Poisson’s ratio predicted by the RoM model are consistent with experimental and finite element results, whereas the transverse and shear moduli tend to be overestimated [131]. This discrepancy primarily arises from the assumption of iso-stress or iso-strain conditions, under which the stress or strain in the fibre, matrix, and composite is considered identical, an assumption that does not accurately represent the actual material behaviour.

Another source of error under transverse loading is the use of the transverse fibre modulus $E_{2f}$ in the formulation, since accurate measurement of the transverse fibre modulus is difficult. This limitation also contributes to inaccuracies in the predicted shear modulus. The elastic moduli obtained from the RoM (Voigt model) and iRoM (Reuss model) provide the upper and lower bounds, respectively [132,133]. Similarly, the shear moduli obtained under iso-shear stress and iso-shear strain conditions provide the lower and upper bounds, respectively. The shear modulus obtained using the higher-order shear stress distribution provides more restrictive lower-bound estimates at higher fibre volume fractions. Therefore, experimental estimates should fall within these bounds.

#### 5.2.3. Hashin Shtrikman Bounds

Hashin and Shtrikman developed narrower and improved bounds in 1963 [134,135], based on the minimum potential energy and variational principles together with the concept of polarization. The strain energy $U_{0}$ of an isotropic and homogeneous reference material is first defined, after which the change in elastic strain energy $U_{p}$ resulting from the addition of inclusions with different elastic properties is considered [136]. The stress polarization tensor *p* is defined as

(26) σ=2Gϵ+λtr(ϵ)I+p

Here, σ and ϵ represent the stress and strain components in the body containing inclusions, while λ and *G* denote the Lamé constant and shear modulus, respectively. *I* represents the identity matrix and tr(·) denotes the trace operator. The change in elastic strain energy is maximized with respect to the stress polarization tensor to determine the bounds. The upper bound is obtained when the reference material possesses higher moduli than the other constituent, whereas the lower bound is obtained when the reference material has lower moduli. These bounds are formulated using the effective elastic properties of the constituent materials rather than solely their volume fractions. For the lower bound of the effective bulk modulus $\left(K_{HSL}\right)$ and shear modulus $\left(G_{HSL}\right)$, the matrix is selected as the reference material. Conversely, for the upper bounds of the effective bulk modulus $\left(K_{HSU}\right)$ and shear modulus $\left(G_{HSU}\right)$, the fibre is selected as the reference material. The lower $\left(K_{HSL}\right)$ and upper $\left(K_{HSU}\right)$ bounds of the effective bulk modulus are given by

(27) $$K_{HSL}=K_{m}+\frac{V_{f}}{\left[{\left(K_{f}-K_{m}\right)}^{-1}+V_{m}{\left(K_{m}+\frac{4}{3}\mu_{m}\right)}^{-1}\right]}$$

(28) $$K_{HSU}=K_{f}+\frac{V_{m}}{\left[{\left(K_{m}-K_{f}\right)}^{-1}+V_{f}{\left(K_{f}+\frac{4}{3}\mu_{f}\right)}^{-1}\right]}$$

The lower $\left(G_{HSL}\right)$ and upper $\left(G_{HSU}\right)$ bounds of the effective shear modulus are expressed as

(29) $$G_{HSL}=G_{m}+\frac{V_{f}}{\left[{\left(G_{f}-G_{m}\right)}^{-1}+\frac{6V_{m}\left(K_{m}+2G_{m}\right)}{5G_{m}\left(3K_{m}+4G_{m}\right)}\right]}$$

(30) $$G_{HSU}=G_{f}+\frac{V_{m}}{\left[{\left(G_{m}-G_{f}\right)}^{-1}+\frac{6V_{f}\left(K_{f}+2G_{f}\right)}{5G_{12f}\left(3K_{f}+4G_{f}\right)}\right]}$$

where $K_{f},G_{f},K_{m}$, and $G_{m}$ denote the bulk and shear moduli of the fibre and matrix, respectively, while $V_{f}$ and $V_{m}$ represent the corresponding volume fractions of the fibre and matrix. The bounds for the elastic modulus can be calculated from the bulk and shear moduli using

(31) $$E=\frac{9KG}{3K+G}$$

These bounds provide a relatively simple but useful means of estimating the approximate effective mechanical properties. The upper and lower bounds converge when the fibre volume fraction is either zero or unity.

#### 5.2.4. Chamis Model

The Rule of Mixtures formulations (Equations (17)–(21)) are obtained without accounting for a specific fibre-packing geometry. The inaccuracies in the transverse and shear moduli predicted by Equations (18) and (20) may be partly attributed to their dependence on the fibre-packing arrangement. Hopkins and Chamis [137] proposed a model for predicting the transverse and shear moduli based on a square fibre-packing arrangement and by subdividing the RVE into several regions. In this approach, the circular fibre cross-section is represented by a square cross-section. The sub-region A consists entirely of matrix material, whereas sub-region B contains both fibre and matrix. By equating the area of the circular fibre to that of the equivalent square fibre, the volume fraction of the circular fibre can be determined.

Using this relationship in the iRoM and combining the effective elastic moduli associated with the three sub-regions gives

(32) $$E_{22}=E_{m}\left[\left(1-\sqrt{V_{f}}\right)+\frac{\sqrt{V_{f}}}{1-\sqrt{V_{f}}\left(1-\frac{E_{m}}{E_{22f}}\right)}\right]$$

Chamis further proposed simplified expressions for the transverse elastic and shear moduli, which are given by [138,139]

(33) $$E_{22}=\frac{E_{m}}{1-\sqrt{V_{f}}\left(1-\frac{E_{m}}{E_{22f}}\right)}$$

(34) $$G_{ij}=\frac{G_{m}}{1-\sqrt{V_{f}}\left(1-\frac{G_{m}}{G_{ijf}}\right)},\;i,j=1,2or2,3$$

The simplified Chamis formulations provide more accurate predictions than the original formulation given in Equation (32). Furthermore, the model provides a more restrictive prediction of the transverse elastic modulus compared with the RoM model. The predictions obtained using the Chamis model were compared with finite-element-based simulations [139]. Jacquet et al. [140] extended the RoM approach by adopting a similar concept to that of the Chamis model for different fibre-packing geometries. The shear modulus predictions obtained from these models are also satisfactory. However, a limitation of the Chamis model is its fundamental assumption of a square fibre cross-section, although it provides a more stringent lower bound than the Reuss model.

#### 5.2.5. Micromechanical Modelling of Effective Elastic Properties

A micromechanical approach can be used to predict the effective elastic properties of two-phase polymer composites by considering the properties and volume fractions of the matrix and reinforcement. The modelling procedure is based on the Eshelby theory, while the Mori–Tanaka, Tandon–Weng, and related formulations can be used to determine the effective elastic constants of fibre- or particle-reinforced isotropic matrix composites [141,142]. The formulation provides the effective Young’s moduli, shear moduli, bulk modulus, and Poisson’s ratio of the composite.

The longitudinal Young’s modulus of the composite can be expressed as [109]

(35) $$\frac{E_{11}}{E_{m}}=\frac{1}{1+\frac{c_{r}\left(A_{1}+2\nu_{m}A_{2}\right)}{A}},$$

while the transverse Young’s moduli are given by

(36) $$\frac{E_{22}}{E_{m}}=\frac{E_{33}}{E_{m}}=\frac{1}{1+\frac{c_{r}-2\nu_{m}A_{3}+\left(1-\nu_{m}\right)A_{4}+\left(1+\nu_{m}\right)A_{5}}{2A}}.$$

The effective shear moduli in the principal directions can be determined from

(37) $$\frac{\mu_{12}}{\mu_{m}}=1+\frac{c_{r}}{2c_{m}S_{1212}+\frac{\mu_{m}}{\mu_{r}-\mu_{m}}},$$

and

(38) $$\frac{\mu_{23}}{\mu_{m}}=1+\frac{c_{r}}{2c_{m}S_{2323}+\frac{\mu_{m}}{\mu_{r}-\mu_{m}}}.$$

The effective bulk modulus in the transverse plane is expressed as

(39) $$\frac{\kappa_{23}}{\kappa_{m}}=\frac{\left(1+\nu_{m}\right)\left(1-2\nu_{m}\right)}{1-\nu_{m}\left(1+2\nu_{12}\right)+\frac{c_{r}\left[2\left(1-\nu_{12}\right)A_{3}+A_{4}\left(1-\nu_{m}\left(1+2\nu_{12}\right)\right)\right]}{A}}.$$

The corresponding Poisson’s ratio is obtained from

(40) $$\nu_{12}=\frac{\nu_{m}A-c_{r}\left(A_{3}-\nu_{m}A_{4}\right)}{A+c_{r}\left(A_{1}+2\nu_{m}A_{2}\right)}.$$

where $E_{ij}$ denotes Young’s modulus in the corresponding material direction; $c_{r}$ is the volume fraction of the reinforcement; and $A_{1}$, $A_{2}$, $A_{3}$, $A_{4}$, $A_{5}$, and *A* are scale factors associated with the reinforcement geometry and elastic properties. The parameters $\nu_{m}$ and $\nu_{r}$ represent the Poisson’s ratios of the matrix and reinforcement, respectively, while $\mu_{m}$ and $\mu_{r}$ denote their corresponding shear moduli. The terms $S_{1212}$ and $S_{2323}$ represent components of the Eshelby tensor, and $\kappa_{m}$ and $\kappa_{23}$ denote the matrix and effective volumetric moduli, respectively.

The expressions for the scale factors $A_{1}$, $A_{2}$, …, $A_{5}$, and *A* and the Eshelby tensor components depend on the geometry of the inclusions and the elastic characteristics of the constituent phases [110]. These relations provide a mathematical means of determining the effective macroscopic elastic properties of the composite from the properties of its constituent materials. For structural analysis, the resulting effective properties can subsequently be combined with appropriate equivalent-stress criteria and failure criteria, such as the Tsai–Hill criterion, to evaluate the overall strength of the composite structure [143,144].

The material properties used in mathematical models can also vary considerably with ageing time. Figure 4 illustrates the dependence of the mean yield strength and its standard deviation on ageing time for BSPE 22007-16 polymer material exposed to cold climate conditions. The results demonstrate that both the mean strength and the associated statistical variation change with increasing ageing time, highlighting the importance of considering material degradation and statistical variability when selecting material parameters for mathematical modelling [109].

The numerical simulation of composite materials provides an additional approach for evaluating the influence of reinforcement content on their mechanical response. In the study considered, MSC Digimat MF was used to simulate the behaviour of polycarbonate-based composites containing different volume fractions of glass-fibre inclusions. The modelling results indicated that increasing the glass-fibre content resulted in higher ultimate strength, modulus of elasticity, and overall material reliability. The corresponding simulated stress–strain responses are presented in Figure 5.

As shown in Figure 5, increasing the inclusion content produces a progressive increase in the predicted stress response. The composite containing 30% inclusions exhibits the highest stress level, followed by the 20% and 10% inclusion cases. This trend demonstrates the ability of the numerical model to describe the effect of reinforcement content on the mechanical response of the polymer composite.

The key assumptions, applicability, calibration requirements, and predictive limitations of the micromechanical and homogenization models reviewed above are summarized in Table 7.

## 6. Continuum-Scale Modelling of Polymer Composites

Continuum-scale models commonly represent polymer nanocomposites using effective-medium descriptions, where nanofillers and their surrounding interphase are incorporated into constitutive parameters for structural analysis. Classical homogenization approaches, including the rule of mixtures [148], Halpin–Tsai relations [149], Mori–Tanaka [146], self-consistent schemes [147], and Eshelby-based inclusion models [145], are widely used as baseline models and extended to account for filler geometry, orientation, imperfect interfaces, and interphase effects. However, reinforcement in polymer nanocomposites is strongly influenced by interfacial physics and morphology, which are not fully captured by phenomenological effective-property models.

An important approach is the explicit representation of the interphase, a region surrounding the nanoparticle with properties different from the bulk polymer. This can be achieved through core-shell inclusion models or interphase-augmented micromechanics, where interphase thickness and stiffness are introduced as additional parameters. Recent studies have examined the coupled effects of interphase size and stiffness on effective modulus, enabling interphase effects to be incorporated into micromechanical predictions [150]. Similarly, molecular-informed constitutive models use molecular simulations to derive continuum-scale parameters, allowing chemistry- and interface-dependent behaviour to be incorporated into macroscopic descriptions [151].

Continuum models provide high computational efficiency and are readily compatible with structural finite-element analysis. However, their accuracy depends on assumptions regarding filler dispersion, interphase characteristics, and interfacial mechanics. When nanoparticle networks, agglomeration, or preferential filler alignment govern the macroscopic response, a single effective-modulus formulation may be inadequate without incorporating morphological descriptors or multiscale-informed constitutive parameters.

## 7. Viscoelasticity and Viscoplasticity

The time-dependent mechanical response of polymer matrix composites (PMCs) is strongly influenced by the viscoelastic and viscoplastic characteristics of the polymer matrix. Under elevated stresses, temperatures, and matrix-dominated loading conditions, PMCs may exhibit both recoverable time-dependent deformation and irreversible strain accumulation.

Viscoelasticity is primarily associated with rate-dependent phenomena such as creep and stress relaxation, whereas viscoplasticity describes the development of permanent strains that depend on the loading history [152,153]. The relative contribution of these mechanisms is highly dependent on the loading direction and the constituent architecture. In many fibre-reinforced PMCs, the response along the fibre direction remains predominantly linear elastic up to failure because of the high stiffness of the reinforcing fibres, whereas nonlinear and time-dependent effects become more significant under transverse and shear loading, where the polymer matrix and fibre–matrix interface exert greater influence.

Experimental studies have reported pronounced nonlinear stress–strain responses in these matrix-dominated directions, particularly at higher strain levels and loading rates [152]. These observations highlight the need for constitutive formulations capable of incorporating strain-rate dependence, time-dependent deformation, and irreversible material evolution when predicting the mechanical response of polymer-based composite systems.

## 8. Advanced Fracture and Damage Modelling

### 8.1. Continuum Damage Mechanics Formulation

Spahn et al. [154] employed a strain-based continuum damage model based on the formulation proposed by Simo and Ju [155] to represent matrix fracture. Within this framework, the damage formulation is used specifically to capture matrix degradation and failure, while the mechanical response of the individual microscopic constituents can be described using other appropriate constitutive models. This modularity allows the damage formulation to be incorporated into broader multiscale modelling frameworks without restricting the constitutive description of the constituent phases.

In the framework of continuum damage mechanics (CDM), Kachanov [156] introduced an internal damage variable *d*, bounded between 0 and 1. The limiting value d=0 corresponds to an undamaged material, whereas d=1 represents complete material failure. The constitutive response can be formulated in terms of the Helmholtz free-energy potential, which, for the present damage formulation, is expressed as

(41) $$\psi =\frac{1}{2}\left(1-d\right)\epsilon :C_{\mathrm{el}}:\epsilon .$$

The Cauchy stress tensor is consequently obtained from the derivative of the free energy with respect to the strain tensor as

(42) $$\sigma =\frac{𝜕\psi }{𝜕\epsilon }=\left(1-d\right)C_{\mathrm{el}}:\epsilon ,$$

where $C_{\mathrm{el}}$ denotes the isotropic elastic stiffness tensor. The thermodynamic force conjugate to the damage variable, *Y*, is determined from the derivative of the Helmholtz free energy with respect to *d* and is given by

(43) $$Y=-\frac{𝜕\psi }{𝜕d}=\frac{1}{2}\epsilon :C_{\mathrm{el}}:\epsilon .$$

For isothermal processes, the second law of thermodynamics is enforced through the Clausius–Duhem inequality,

(44) $$D=P-\dot{\psi }\geq 0,$$

where D denotes the mechanical dissipation and $P=\sigma :\dot{\epsilon }$ represents the rate of mechanical work. Assuming that no additional dissipative mechanisms are present, substitution of the constitutive relations into the Clausius–Duhem inequality yields

(45) $$D=Y\dot{d}\geq 0.$$

The evolution of the damage variable is governed by a damage criterion *f*. For monotonically increasing damage, this criterion can be defined in terms of a monotonic function ϕ(Y) as

(46) $$f\left(Y,d\right)=\phi \left(Y\right)-d\leq 0\;\left\{\begin{array}{cc} f<0 & \mathrm{elastic},\\ f=0 & \mathrm{damage}. \end{array}\right.$$

Combining the damage criterion with the dissipation inequality and invoking the principle of maximum dissipation leads to a constrained optimization problem [155,157]. Introducing the non-negative Lagrange multiplier $\dot{\lambda }$, the corresponding Lagrangian can be written as

(47) $$L=-D+\dot{\lambda }f=-Y\dot{d}+\dot{\lambda }\left(\phi (Y)-d\right).$$

The stationarity conditions associated with this constrained formulation provide the evolution equation for the damage variable. In the active damage regime, where f=0, the damage rate is therefore expressed as

(48) $$\dot{d}=\frac{𝜕f}{𝜕Y}\dot{\lambda }=\frac{𝜕\phi }{𝜕Y}\dot{\lambda }.$$

This formulation provides a thermodynamically consistent description of progressive material degradation and fracture through a single internal damage variable. Its relatively simple structure also makes it suitable for incorporation into multiscale frameworks, although its predictive capability remains dependent on the selected damage criterion and the assumptions underlying the constitutive representation.

### 8.2. Phase-Field Method

The phase-field method (PFM) is a variational approach to fracture modelling that represents a sharp crack by a finite-width, diffuse damage zone, thereby avoiding explicit tracking of crack surfaces and displacement discontinuities [158,159]. A major advantage of PFM is its ability to describe crack initiation, propagation, branching, and merging without prescribing the crack path a priori. This capability makes it particularly attractive for heterogeneous materials such as polymer matrix composites (PMCs), where matrix cracking, fibre fracture, and interfacial debonding may develop simultaneously and interact during damage evolution.

In the conventional phase-field formulation, a continuous scalar field ϕ is introduced to approximate the discrete crack surface, with ϕ=0 representing the intact material and ϕ=1 corresponding to a fully developed crack. The transition between these states is controlled by the characteristic length-scale parameter $l_{0}$, which determines the width of the regularized fracture zone. For a one-dimensional crack representation, the phase field can be expressed as $\phi \left(x\right)=\exp(-|x|/l_{0})$, indicating that the sharp crack is replaced by a smoothly varying damage profile [160]. The corresponding crack-surface density function can be written as

(49) $$\gamma \left(\phi ,\nabla \phi \right)=\frac{1}{2l_{0}}\left(\phi^{2}+l_{0}^{2}{\left|\nabla \phi \right|}^{2}\right).$$

Here, the first term represents the local contribution of the phase field, whereas the gradient term governs the spatial regularization of the crack. Consequently, the fracture energy is distributed over a finite region surrounding the crack rather than being concentrated on an idealized discontinuous surface.

Following the variational framework proposed by Francfort and Marigo [161], the equilibrium state of a quasi-static brittle fracture problem is obtained by minimizing the total potential energy,

(50) $$\Pi =\Pi_{e}+\Pi_{g}-\Pi_{\mathrm{ext}},$$

where $\Pi_{e}$ is the elastic strain energy, $\Pi_{g}$ is the regularized fracture energy, and $\Pi_{\mathrm{ext}}$ represents the potential of the external forces. The fracture contribution is commonly expressed as

(51) $$\Pi_{g}=\int_{\Omega }G_{c}\left(\frac{\phi^{2}}{2l_{0}}+\frac{l_{0}}{2}{\left|\nabla \phi \right|}^{2}\right)dV,$$

where $G_{c}$ denotes the critical fracture energy and Ω represents the material domain. The potential of the external loading is given by

(52) $$\Pi_{\mathrm{ext}}=\int_{\Omega }b\cdot u\;dV+\int_{𝜕\Omega }f\cdot u\;dA,$$

where b and f denote the body force and surface traction, respectively, while u is the displacement field.

For composite structures, the principal advantage of PFM is its ability to describe complex fracture evolution within a unified variational framework. By incorporating appropriate constitutive and fracture-energy descriptions for the different constituents, the method can be extended to represent matrix cracking, fibre failure, and interfacial damage, as well as their interactions. However, its predictive accuracy depends strongly on the selection of the degradation function, length-scale parameter, crack-driving force, and constituent-specific fracture energies. In particular, the regularization length $l_{0}$ introduces a characteristic length scale that must be selected carefully in relation to the microstructural dimensions of the composite. Thus, although PFM eliminates the need for explicit crack tracking, its application to heterogeneous composite materials can remain computationally demanding and may require careful calibration to minimize mesh- and parameter-dependent effects.

### 8.3. Cohesive Zone Model

The cohesive zone model (CZM) is a widely established fracture framework for describing progressive separation across material interfaces. In composite structures, it is particularly effective for modelling interlaminar delamination, adhesive joint failure, and fibre/matrix interfacial debonding, where fracture develops through the gradual degradation of interfacial load transfer rather than through an initially sharp crack.

Unlike approaches that explicitly track crack surfaces, CZM introduces an interface constitutive law that relates traction to separation and allows damage initiation and subsequent degradation to be represented within a unified formulation. Its relatively straightforward implementation in finite element models has contributed to its widespread use in composite fracture analysis.

The fundamental assumption of CZM is that the fracture process zone can be represented by a traction–separation relationship that characterizes the progressive loss of cohesive strength at an interface. Several constitutive forms have been proposed, including bilinear [162], trapezoidal [163], linear-parabolic [164], and exponential laws [165].

Although these formulations differ in the representation of the softening response, they generally describe fracture through two successive stages: damage initiation, associated with the attainment of the interface strength, and damage evolution, characterized by the progressive reduction of traction with increasing separation.

The bilinear traction–separation law is among the most commonly adopted formulations because of its simplicity and its ability to reproduce the essential characteristics of progressive interfacial failure. Under pure mode I, II, or III loading, the interface initially responds elastically, with the traction increasing linearly with separation according to the penalty stiffness $K_{i}$. Damage is initiated when the corresponding peak traction $\sigma_{\max}$ is reached.

Beyond this point, the interface enters a softening regime in which the traction decreases with increasing separation until it vanishes at the critical separation. The associated fracture energy is given by the area under the traction–separation curve and corresponds to the critical energy release rate $G_{c}$. Accordingly, the displacement at damage initiation and the displacement corresponding to complete failure can be expressed as [166]

(53) $$\delta^{0}=\frac{\sigma_{\max}}{K},\;\delta^{f}=\frac{2G_{c}}{\sigma_{\max}}.$$

For each fracture mode i=I,II,III, the bilinear cohesive law can consequently be written as [167]

(54) $$\sigma_{i}=\left\{\begin{array}{cc} K_{i}\delta_{i}, & \delta_{i}\leq \delta_{i}^{0},\\ \left(1-d_{i}\right)K_{i}\delta_{i}, & \delta_{i}>\delta_{i}^{0}, \end{array}\right.$$

where $K_{i}$ is the penalty stiffness, $\delta_{i}$ is the separation displacement, and $d_{i}$ is the scalar damage variable. The evolution of damage between its initiation and complete failure states is commonly defined as

(55) $$d_{i}=\frac{\delta_{i}^{f}\left(\delta_{i}-\delta_{i}^{0}\right)}{\delta_{i}\left(\delta_{i}^{f}-\delta_{i}^{0}\right)},\;i=I,II,III,\;d_{i}\in \left[0,1\right].$$

Here, $d_{i}=0$ corresponds to an undamaged interface, whereas $d_{i}=1$ represents complete loss of cohesive load-carrying capacity. The formulation therefore provides a convenient means of linking experimentally measurable interface properties, such as cohesive strength and fracture energy, with the numerical representation of progressive delamination.

For mode-I loading, an additional contact condition is required to prevent nonphysical penetration of the opposing crack faces. When the normal separation becomes negative, the interface is considered to be in compression and the tensile damage law is no longer active. The normal traction can then be represented as

(56) $$\sigma_{I}=K_{I}\delta_{I},\;\delta_{I}\leq 0.$$

This treatment ensures that crack closure generates a compressive reaction while preventing the cohesive surfaces from interpenetrating. In mixed-mode formulations, Macaulay brackets 〈·〉 are commonly introduced so that only positive normal separation contributes to tensile damage, whereas negative values are interpreted as contact and do not promote crack growth [167,168].

Despite its computational efficiency and relatively simple implementation, CZM has important limitations when applied to complex composite architectures. The location of the cohesive interface must generally be specified in advance, which can restrict its ability to predict arbitrary crack paths or unexpected damage locations.

Moreover, the predicted response is sensitive to the selected cohesive strength, penalty stiffness, fracture energy, and shape of the traction–separation law. These parameters are often difficult to determine independently from experiments, and their calibration may substantially influence the predicted initiation and propagation of delamination.

The use of an excessively large penalty stiffness can also introduce numerical ill-conditioning, whereas an excessively low value may produce artificial compliance. Consequently, CZM is particularly suitable when the potential fracture interfaces are known a priori, but its applicability becomes more limited for heterogeneous composites in which multiple competing damage mechanisms and unconstrained crack paths must be considered.

The key features, assumptions, applicability, and limitations of these continuum damage and fracture models are summarized in Table 8.

## 9. Numerical Modelling of Polymer Hybrid Materials

Numerical modelling provides an efficient approach for analysing and predicting the mechanical response of polymer hybrid materials, particularly when complex material architectures and loading conditions make extensive experimental investigation difficult.

Analytical and numerical studies have been applied to a range of composite and hybrid systems, demonstrating their potential for reducing computational time and supporting structural design [169,170,171,172].

Several numerical approaches are available for composite-material analysis. The finite element method (FEM) has been widely employed for progressive damage and multiscale analysis of composite laminates [173,174], while the finite difference method (FDM) has been applied to layered composite structures [175]. Boundary element methods (BEMs) provide an alternative formulation for composite-material and fibre-reinforced composite analysis [176,177,178]. Discrete approaches have also been applied to structural and discontinuous problems, including laminated composite vibration and buckling analyses and discrete element modelling of contact behaviour [179,180].

Despite the versatility of these approaches, their predictive reliability depends strongly on the underlying constitutive assumptions, material-property representation, interface modelling, and damage criteria. Therefore, the selection of an appropriate numerical formulation and its validation against experimental data remain essential for developing reliable predictive models of polymer hybrid materials. The principal numerical approaches considered in this review are presented in the flowchart in Figure 6.

### 9.1. Finite Difference Method (FDM)

The finite difference method (FDM) provides a grid-based numerical framework for solving governing differential equations by approximating spatial derivatives at discrete nodal points. Its relatively simple formulation makes it attractive for composite-material problems, although complex geometries, material heterogeneity, and anisotropy may require appropriate grid treatment. The application of numerical modelling to hybrid composite materials has been discussed by Thakur et al. [92] and Bouhfd et al. [181].

For small deformations, the displacement field $u_{i}$ is related to the infinitesimal strain tensor by

(57) $$\varepsilon_{ij}=\frac{1}{2}\left(\frac{𝜕u_{i}}{𝜕x_{j}}+\frac{𝜕u_{j}}{𝜕x_{i}}\right).$$

For an isotropic linear-elastic material, the constitutive relation is expressed in terms of the Lamé constants as

(58) $$\sigma_{ij}=\lambda \varepsilon_{kk}\delta_{ij}+2\mu \varepsilon_{ij},$$

where λ and μ are the Lamé constants and $\delta_{ij}$ is the Kronecker delta. In terms of Young’s modulus *E* and Poisson’s ratio ν,

(59) $$\mu =\frac{E}{2(1+\nu )},\;\lambda =\frac{E\nu }{\left(1+\nu )(1-2\nu \right)}.$$

Substitution of these relations into the constitutive equation gives

(60) $$\sigma_{ij}=\frac{E}{1+\nu }\left[\varepsilon_{ij}+\frac{\nu }{1-2\nu }\delta_{ij}\varepsilon_{kk}\right].$$

The equilibrium condition for a continuum subjected to a body-force vector $f_{i}$ is obtained from force balance as

(61) $$\frac{𝜕\sigma_{ij}}{𝜕x_{j}}+f_{i}=0.$$

For FDM implementation, the spatial derivatives are then discretized over the computational grid. For example, the first derivative at an interior node can be approximated using the central-difference scheme,

(62) $${\left.\frac{𝜕u}{𝜕x}\right|}_{i}\approx \frac{u_{i+1}-u_{i-1}}{2\Delta x}.$$

The resulting algebraic system can subsequently be solved for the displacement, strain, and stress fields. For polymer hybrid materials, however, the basic isotropic formulation must be extended to account for anisotropic reinforcement, heterogeneous interfaces, and material-specific constitutive behaviour [175,181].

### 9.2. Finite Element Method (FEM)

The finite element method (FEM) is widely employed for analysing polymer hybrid materials because of its flexibility in representing complex geometries, reinforcement arrangements, material interfaces, and heterogeneous mechanical properties. The discretization of a continuous structure into a finite number of elements makes FEM particularly suitable for composite structures with complex geometries and material configurations [182,183].

Following the finite element formulation reported by Riva and Nicoletto [183], the continuous structure is discretized into a finite number of smaller elements connected through nodes. Within each element, the displacement field is approximated using appropriate interpolation or shape functions as

(63) $$u^{\left(m\right)}=N^{\left(m\right)}\delta^{\left(m\right)},$$

where $u^{\left(m\right)}$ denotes the displacement field within element *m*, $N^{\left(m\right)}$ is the shape-function matrix, and $\delta^{\left(m\right)}$ represents the corresponding nodal displacement vector. The element formulation leads to an algebraic relationship between the stiffness matrix, nodal displacement vector, and external force vector:

(64) $${\left[k\right]}_{m}{\left[\delta \right]}_{m}={\left[F\right]}_{m}.$$

By assembling the contributions from all elements, the global finite element equilibrium equation is obtained as

(65) $$\sum_{m}{\left[k\right]}_{m}{\left[\delta \right]}_{m}=\left[F\right].$$

Equation (65) represents the fundamental algebraic form of the finite element formulation, where *m* denotes the element number, ${\left[k\right]}_{m}$ is the element stiffness matrix, ${\left[\delta \right]}_{m}$ is the nodal displacement vector, and [F] represents the global external force vector. The formulation can subsequently be extended to account for the heterogeneous reinforcement distribution and material characteristics of polymer hybrid materials. The accuracy of such models depends on appropriate mesh discretization, material-property definition, interface representation, and experimental validation.

For hybrid composite modelling, multiscale approaches based on representative volume elements and unit-cell descriptions can additionally be employed to represent microstructural characteristics and reinforcement distributions [8,184].

### 9.3. Boundary Element Method (BEM)

The boundary element method (BEM) is an effective numerical approach for analysing composite structures, particularly for problems involving stress concentrations, fracture, and contact behaviour. Unlike domain-based numerical methods, BEM formulates the governing problem in terms of boundary integral equations, thereby reducing the dimensionality of the computational domain. This feature makes BEM attractive for analysing complex boundary conditions and stress states in composite structures [185]. The method has also been applied to composite materials and fibre-reinforced systems, demonstrating its suitability for evaluating mechanical responses at different structural scales [176].

In the context of the initial-stress formulation, the displacement field is represented through a boundary integral equation. Following the formulation reported for boundary element analysis of fibre-reinforced composites [186], the displacement equation can be expressed as

(66) $$\begin{array}{cc} c_{ij}\left(x^{\prime }\right)u_{j}\left(x^{\prime }\right)= & \int_{\Gamma }U_{ij}\left(x^{\prime },x\right)t_{i}\left(x\right)\;d\Gamma \left(x\right)-\int_{\Gamma }T_{ij}\left(x^{\prime },x\right)u_{i}\left(x\right)\;d\Gamma \left(x\right)\\ \; & +\int_{\Omega }E_{ijk}\left(x^{\prime },X\right)\sigma_{jk}^{p}\left(X\right)\;d\Omega \left(X\right). \end{array}$$

where $U_{ij}$ and $T_{ij}$ represent the fundamental solutions associated with displacement and traction, respectively; $E_{ijk}$ denotes the corresponding fundamental solution; and $\sigma_{jk}^{p}$ represents the plastic stress component. The coefficient $c_{ij}\left(x^{\prime }\right)$ depends on the position of the source point relative to the boundary.

For problems involving plastic deformation, the stress distribution within the domain can be obtained using the corresponding integral formulation. Following the initial-stress approach described for numerical modelling of hybrid composite materials [181], the stress field can be expressed as

(67) $$\begin{array}{cc} \sigma_{ij}\left(x^{\prime }\right)= & \int_{\Gamma }U_{ijk}\left(x^{\prime },x\right)t_{k}\left(x\right)\;d\Gamma \left(x\right)-\int_{\Gamma }T_{ijk}\left(x^{\prime },x\right)u_{k}\left(x\right)\;d\Gamma \left(x\right)\\ \; & +\int_{\Omega }E_{ijkl}\left(x^{\prime },X\right)\sigma_{kl}^{p}\left(X\right)\;d\Omega \left(X\right)+F_{ijkl}\sigma_{kl}^{p}\left(x^{\prime }\right). \end{array}$$

where $U_{ijk}$, $T_{ijk}$, $E_{ijkl}$, and $F_{ijkl}$ denote the corresponding fundamental solutions. Equations (66) and (67) provide the integral representation of displacement and stress fields within the BEM framework. For polymer hybrid materials, the effectiveness of BEM depends on the appropriate representation of heterogeneous constituents, reinforcement distributions, and material interfaces. Consequently, careful formulation of the boundary conditions and material response is essential for obtaining reliable predictions of hybrid composite behaviour.

A comparative assessment of the main numerical methods used for polymer hybrid materials is presented in Table 9.

### 9.4. Molecular Dynamics Modelling

Molecular dynamics (MD) provides an atomistic framework for describing mechanical response, interfacial interactions, and fracture mechanisms in polymer-based composite systems. Unlike continuum approaches, MD resolves molecular interactions between the polymer matrix and reinforcing phase, allowing constituent–matrix load transfer and local failure processes to be examined at the atomistic scale. Kallivokas et al. [187] investigated the interfacial shear behaviour of epoxy–graphene nanocomposites and evaluated interfacial shear strength and associated kinetic and local mechanical parameters for different graphene surface conditions.

For epoxy–CNT nanocomposites, Delasoudas et al. [188] examined interfacial failure from a molecular perspective, relating the applied conditions to local failure mechanisms and their molecular origins. These studies demonstrate the capability of MD to resolve reinforcement–matrix interactions and interfacial mechanisms that are difficult to represent explicitly in conventional continuum formulations.

MD has also been extended from interfacial characterization to fracture-property prediction. Delasoudas et al. [189] developed a scale-bridging atomistic approach for highly cross-linked epoxy networks that combines classical force fields with a bond-breaking treatment and continuum fracture mechanics. The approach was used to estimate the macroscopic fracture energy, $G_{C}$, and stress-intensity factor, $K_{I}$, thereby connecting atomistic fracture mechanisms with continuum-scale fracture parameters.

More recently, Delasoudas et al. [190] extended this methodology to glassy epoxy nanocomposites containing pristine and hydroxyl-functionalized carbon nanotubes. The study demonstrated that interfacial adhesion influences load transfer and fracture behaviour and showed that functionalized CNTs can improve fracture properties relative to pristine CNTs. Thus, MD provides a complementary multiscale modelling route in which atomistic interactions and failure mechanisms can inform the mechanical and fracture behaviour of polymer nanocomposites.

The selection of a mathematical formulation for polymer hybrid materials depends on the characteristic scale, material heterogeneity, failure mechanism, and computational cost.

The FEM (finite element method) employs a weak or variational formulation with element-based shape functions, offering flexibility for complex geometries, heterogeneous constituents, and interfaces, whereas FDM directly approximates spatial derivatives on structured grids and is more efficient for regular domains.

For fracture, XFEM represents discontinuities independently of the mesh, CZM describes progressive failure through traction–separation relations at predefined interfaces, and phase-field modelling represents cracks through a regularized damage field, enabling complex crack evolution without explicit tracking.

Molecular dynamics (MD) resolves fracture through atomistic interactions and bond breaking and is therefore suited primarily to nanoscale constituent and interfacial mechanisms. The principal differences among these approaches are summarized in Table 10.

XFEM is suitable for arbitrary crack paths, CZM for predefined interfaces, and phase-field modelling for interacting cracks, whereas MD resolves atomistic fracture mechanisms [158,159,160,162]. Method selection should therefore reflect the hybrid architecture, governing failure mechanism, required resolution, and computational cost.

## 10. Machine Learning for Modelling Polymer Hybrid Composites

Machine learning (ML) has emerged as an effective approach for modelling polymer composites and nanocomposites, particularly where conventional simulations are computationally demanding. By learning nonlinear relationships from data, ML can accelerate multiscale modelling, provide computationally efficient surrogate models, and facilitate inverse material design. However, its reliability depends on data quality, model validation, physical consistency, and integration with established mechanics-based methods.

### 10.1. Data Requirements and Model Development

Data availability and quality remain major challenges in applying ML to polymer composites and nanocomposites. Materials datasets are often limited, heterogeneous, and expensive to generate, relying on molecular dynamics, coarse-grained simulations, finite-element analyses, experiments, or combinations thereof. For nanocomposites, sufficient coverage of filler type, morphology, dispersion, interfacial properties, and loading conditions is particularly challenging.

Input representation is therefore critical. Common descriptors include composition, filler volume fraction, aspect ratio, orientation, interfacial properties, and RVE-based microstructural statistics [193,194,195]. Field-based inputs, including stress and strain fields or microstructure images, have also been combined with convolutional neural networks (CNNs) and graph-based models to capture spatial correlations [196,197]. Appropriate feature engineering or representation learning is essential to avoid spurious correlations and ensure physically meaningful predictions [198].

Limited data availability has further motivated the use of transfer learning, active learning, and physics-informed regularization to improve data efficiency [199,200,201]. These approaches enable models to exploit prior knowledge or selectively generate new data in regions of high prediction uncertainty [202].

### 10.2. Machine Learning for Surrogate Modelling in Multiscale Frameworks

Machine learning (ML) is increasingly used as a complementary tool within multiscale frameworks to accelerate or approximate computationally expensive physics-based models. Surrogate models are particularly effective in learning the input–output relationships of simulations across atomistic, mesoscale, and continuum scales.

At the atomistic scale, ML surrogates can replace or accelerate ab initio and molecular dynamics calculations while retaining high accuracy. Behler and Parrinello [203] introduced high-dimensional neural network potentials trained on quantum-mechanical data, while subsequent developments such as Gaussian approximation potentials (GAPs) [204] and moment tensor potentials [205] demonstrated substantial computational savings. These approaches retain key physical symmetries within the learned representations.

At the mesoscale, ML surrogates have been applied to approximate microstructure-resolved simulations, particularly RVE-based finite element analyses. Bostanabad et al. [206] and subsequent studies [207,208] showed that ML models can predict effective elastic, plastic, and viscoelastic properties from microstructural descriptors such as phase fraction, aspect ratio, and orientation. Image-based CNN approaches further enable direct prediction of effective properties from voxelized microstructures, avoiding repeated FE simulations [209,210]. Figure 7 schematically illustrates how machine learning (ML) can be integrated throughout a multiscale modelling framework, from atomistic-scale surrogate modelling and descriptor extraction to mesoscale homogenization and continuum-scale constitutive representations.

At the continuum scale, ML surrogates have been used to approximate nonlinear and history-dependent constitutive responses. Early studies by Ghaboussi et al. [211,212] demonstrated the use of neural networks to represent complex constitutive relationships, while recent approaches integrate ML into multiscale $FE^{2}$ frameworks to replace microscale calculations and predict stresses, tangents, or internal variables at substantially lower computational cost [213,214].

Overall, ML surrogate modelling can significantly reduce computational cost while maintaining acceptable accuracy within the training domain. This capability is particularly valuable for design optimization, microstructure tailoring, and large-scale property screening. Thus, ML surrogates are increasingly considered complementary enabling tools that extend physics-based multiscale models rather than direct replacements for them.

The principal characteristics, scales, outputs, and limitations of the major theoretical, computational, experimental, and machine-learning approaches used for polymer hybrid materials are comparatively summarized in Table 11.

## 11. Future Scope

Future research on polymer hybrid materials should focus on improving the predictive capability, reliability, and practical applicability of mathematical modelling approaches. Based on the limitations identified in this review, the following areas require further investigation:

- Integrated multiscale modelling: Develop unified frameworks that link constituent properties and microstructural behaviour with the macroscopic mechanical response of polymer hybrid materials.
- Experimental validation: Establish standardized testing procedures and reliable material datasets for consistent model calibration, validation, and comparison.
- Damage and failure prediction: Improve the prediction of interfacial debonding, damage initiation, crack propagation, delamination, and progressive failure under different loading conditions.
- Material variability and reliability: Incorporate constituent variability, manufacturing effects, fibre distribution, and statistical uncertainty into predictive models.
- Environmental and long-term behaviour: Extend existing models to account for ageing, moisture, temperature, degradation, fatigue, and service-life behaviour.
- Advanced computational approaches: Integrate analytical, micromechanical, numerical, and computational methods to improve the efficiency and accuracy of models for complex hybrid architectures.
- Engineering optimization: Develop predictive frameworks to support the selection of fibre combinations, volume fractions, orientations, stacking sequences, and manufacturing conditions for specific engineering requirements.

Overall, future research should aim to establish robust, validated, and uncertainty-aware modelling frameworks capable of predicting the mechanical behaviour, damage evolution, degradation, and structural performance of polymer hybrid materials under practical engineering conditions.

## 12. Conclusions

This review has critically examined the mathematical and computational modelling of polymer hybrid materials, encompassing their classification, mechanical characteristics, and predictive frameworks. Polymer hybrid materials, categorized by reinforcement source, fibre architecture, and structural configuration, achieve enhanced mechanical performance through constituent synergy, though their response remains contingent upon material composition, interfacial bonding, manufacturing conditions, and loading environment.

Hybridization consistently improves tensile, flexural, and impact properties; however, the extent of improvement varies considerably with fibre type, content, treatment, stacking sequence, and testing protocols. This variability, compounded by the inherent heterogeneity of natural fibres and complex constituent interactions, precludes direct quantitative comparisons across studies.

Multiple modelling approaches exist for predicting material behaviour. Analytical and micromechanical models offer computationally efficient property estimates but are constrained by simplifying assumptions. Numerical methods enable detailed analyses of complex geometries, while advanced damage frameworks provide thermodynamically consistent descriptions of progressive failure and interfacial debonding. Machine learning has emerged as a complementary surrogate modelling tool, though its efficacy depends on the availability of high-quality training data.

No single methodology fully captures the multi-scale mechanisms governing hybrid systems. Persistent discrepancies between theoretical, numerical, and experimental results underscore the need for standardized databases, rigorous calibration, and comprehensive validation. Predictive models capable of describing stiffness, stress transfer, damage evolution, and energy absorption remain comparatively underdeveloped.

Greater integration of experimental characterization with analytical and computational modelling is essential for establishing reliable predictive frameworks. Such developments will enhance predictive fidelity, facilitate design optimization, and accelerate the adoption of polymer hybrid materials in engineering applications.

## Figures and Tables

**Figure 1:**
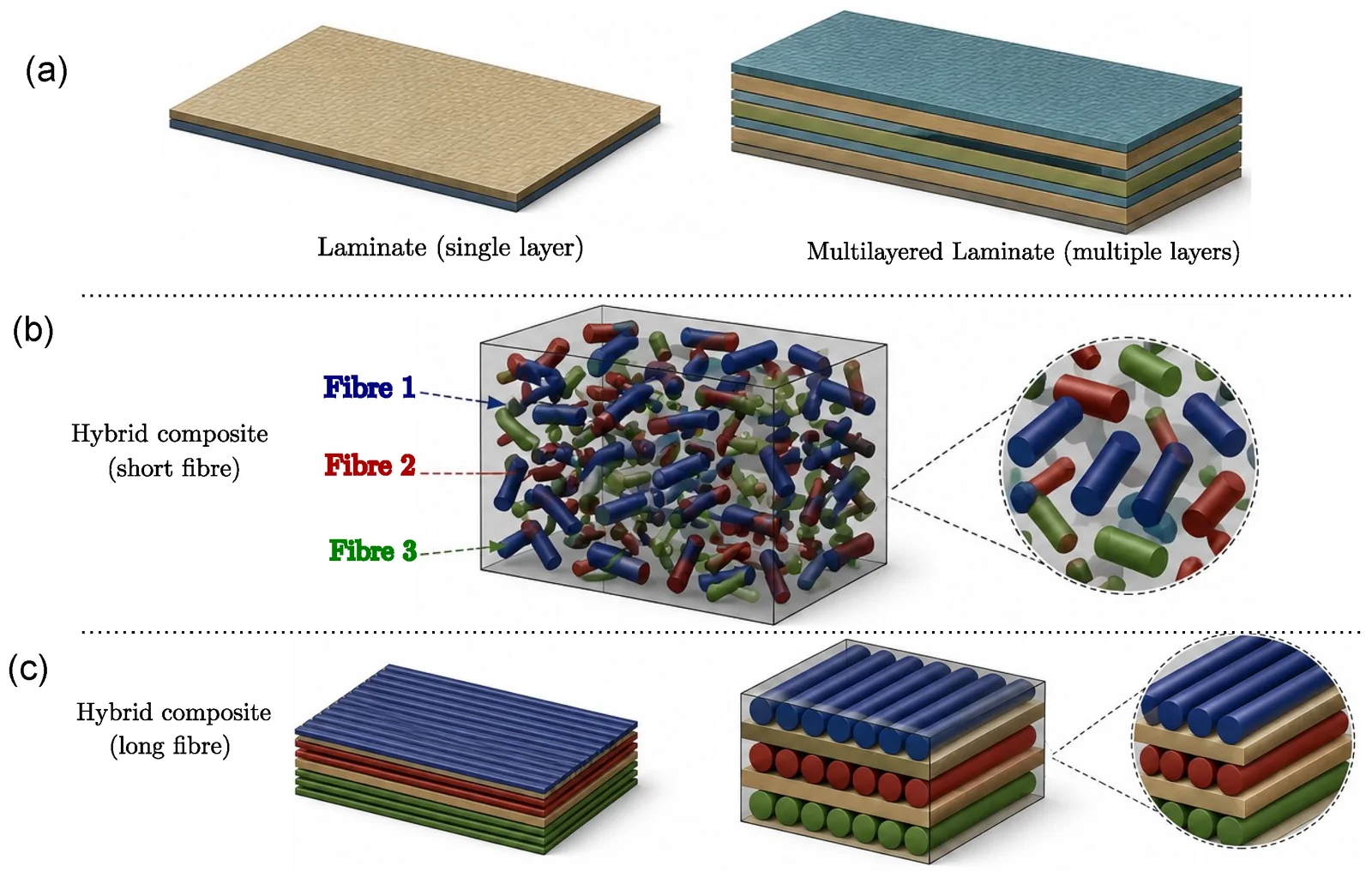
Schematic representation of the principal architectures of polymer hybrid materials: (**a**) laminate and multilayered laminate configurations, (**b**) hybrid composites reinforced with short fibres, and (**c**) hybrid composites reinforced with long fibres.

**Figure 2:**
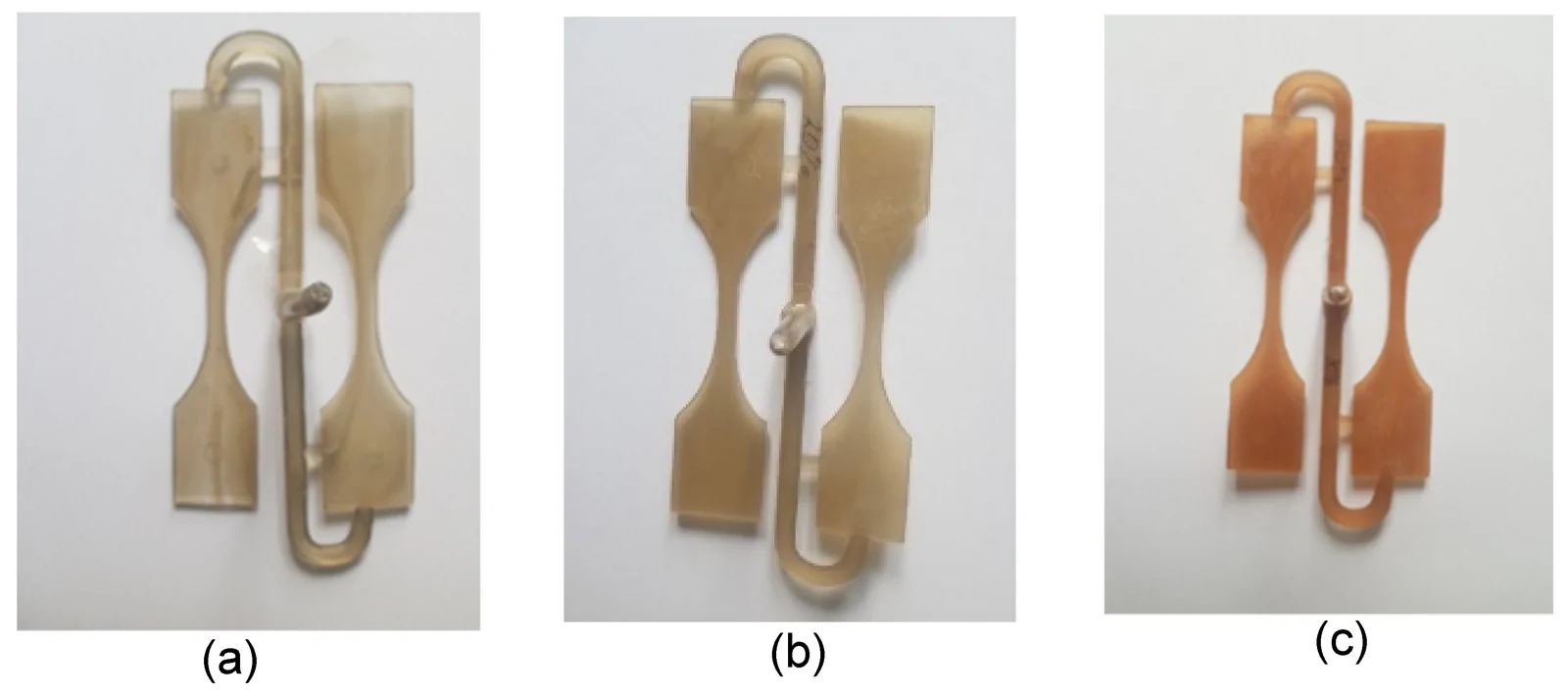
Test specimens of the investigated polymer composites: (**a**) unfilled composite, (**b**) composite containing 20% short glass fibres, and (**c**) composite containing 30% short glass fibres [109].

**Figure 3:**
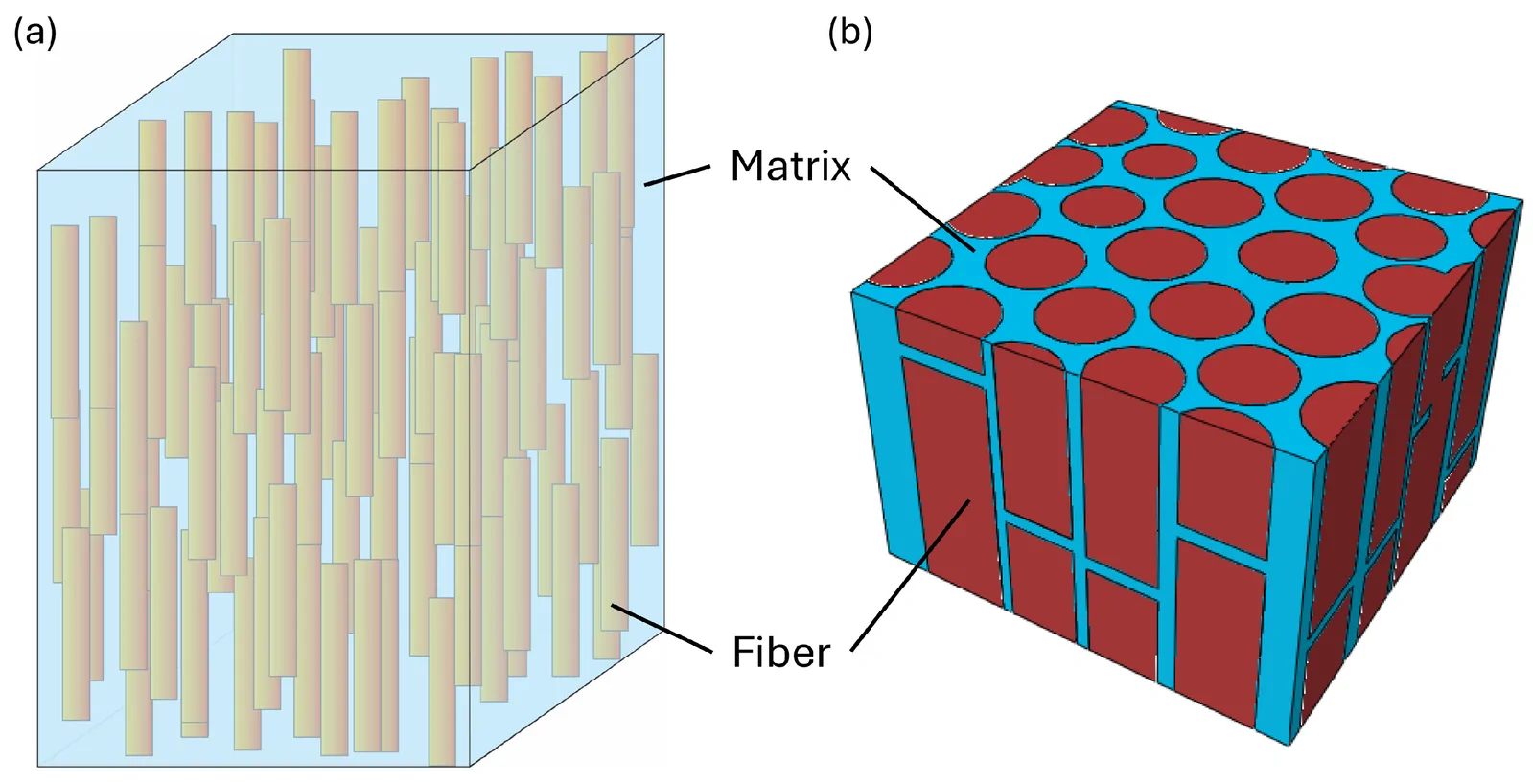
(**a**) Schematic illustration of a polymer composite reinforced with short cylindrical fibres. (**b**) RVE of the polymer composite used for finite-element analysis [119].

**Figure 4:**
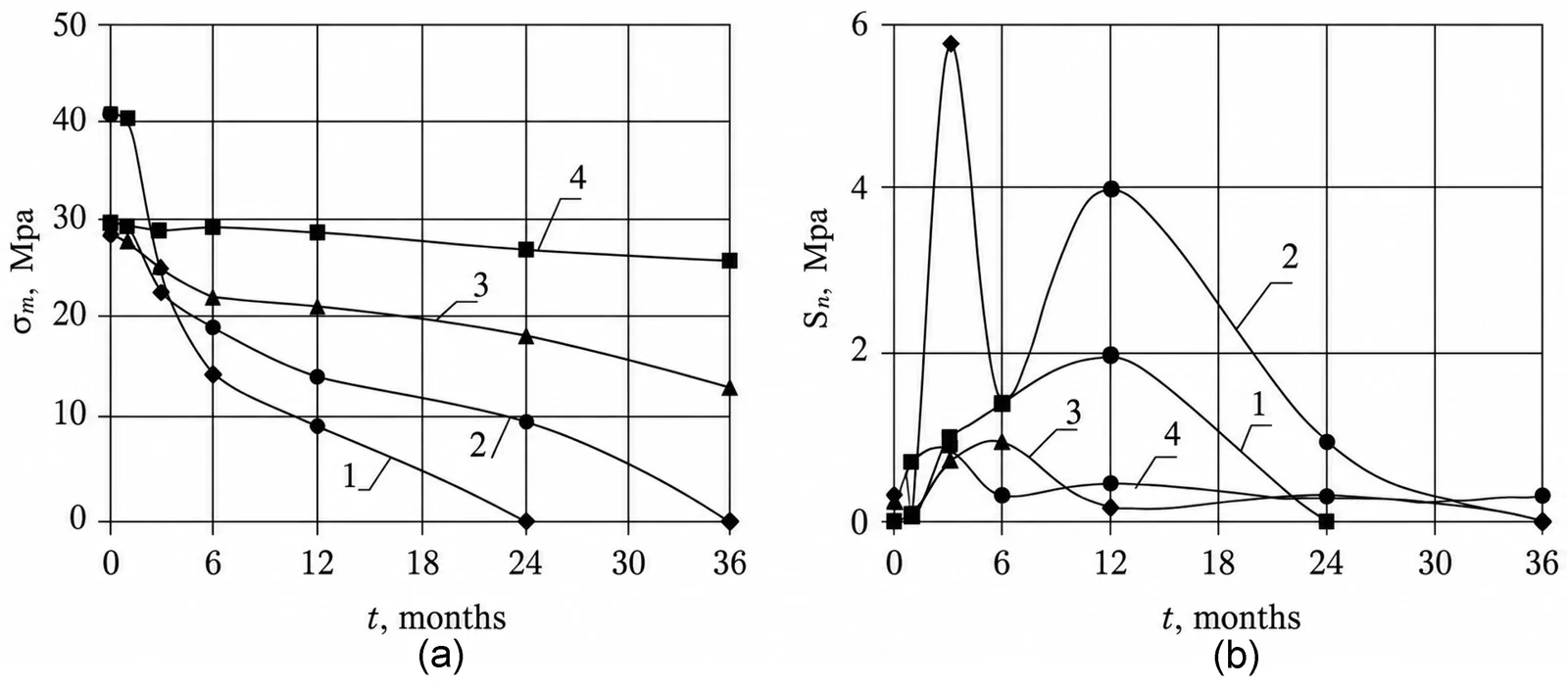
Dependence of (**a**) the mean value and (**b**) the standard deviation of yield strength on ageing time under cold climate conditions for the BSPE 22007-16 material. The cross-sectional areas of the samples are (1) 1×2.5, (2) 2×5, (3) 3×13, and (4) 8×25 ${\mathrm{mm}}^{2}$ [109].

**Figure 5:**
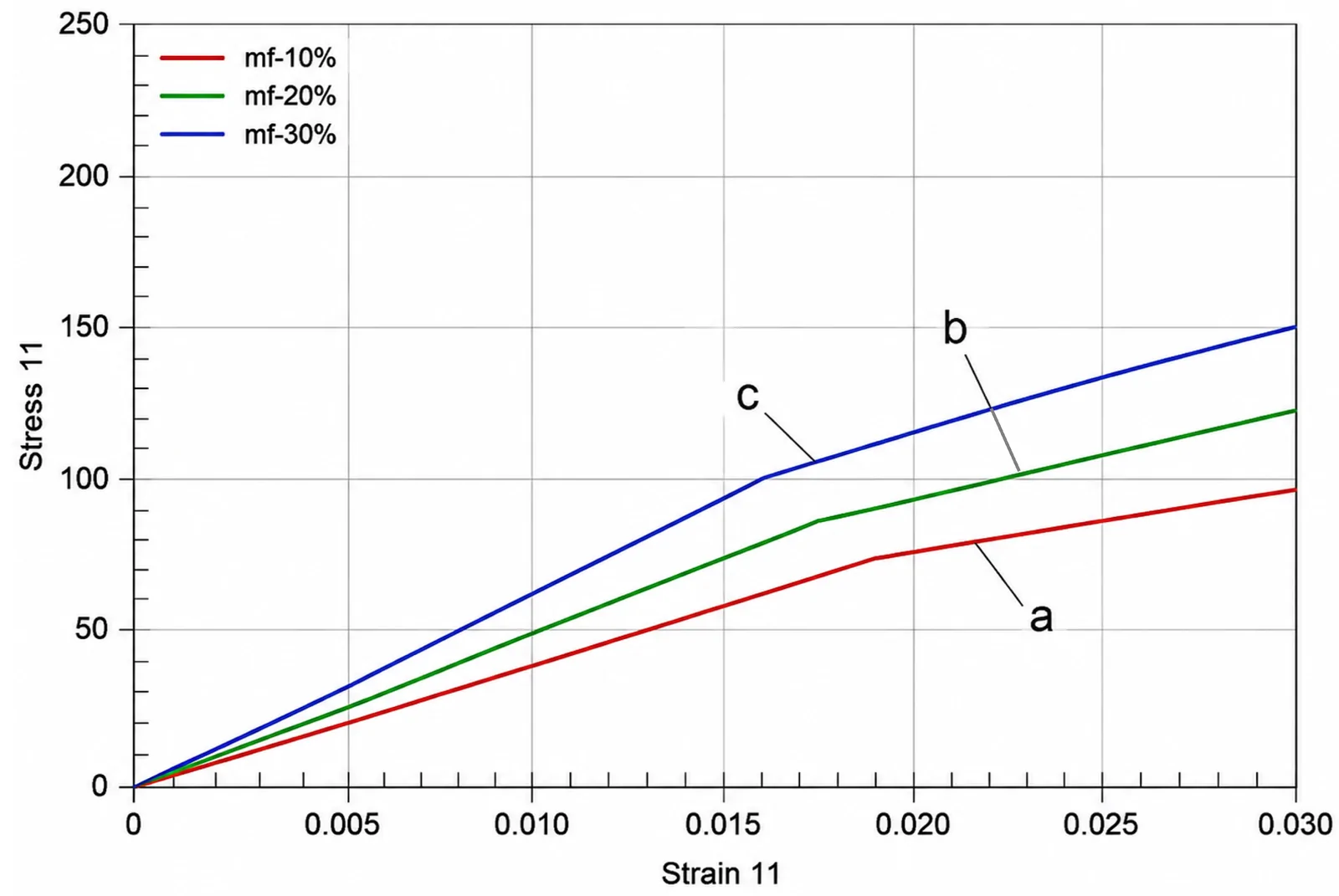
Simulated stress–strain responses of a polycarbonate-based composite containing different inclusion contents: (a) 10% inclusions (red line), (b) 20% inclusions (green line), and (c) 30% inclusions (blue line) [109].

**Figure 6:**
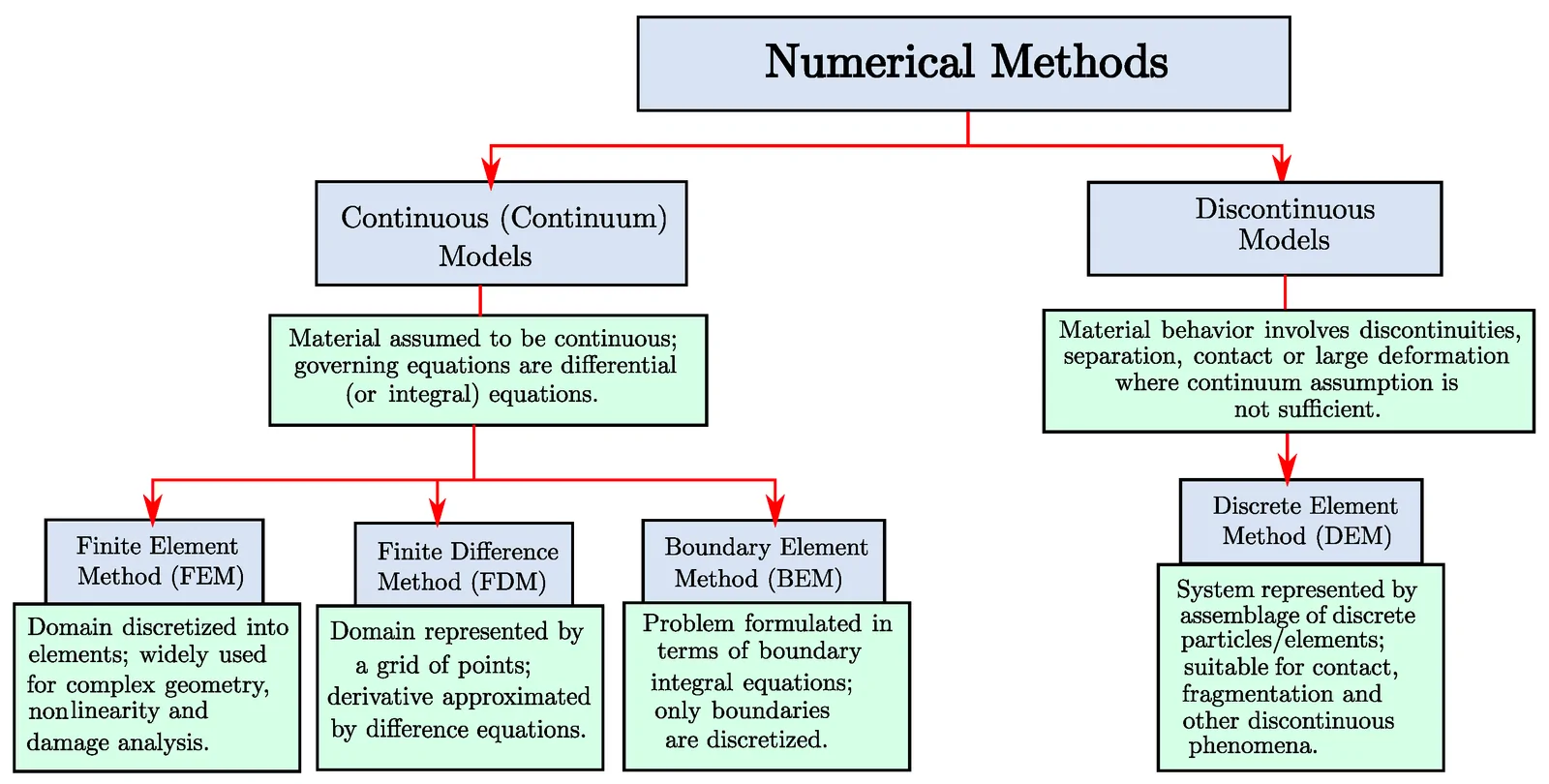
Classification of numerical methods used for modelling polymer hybrid materials.

**Figure 7:**
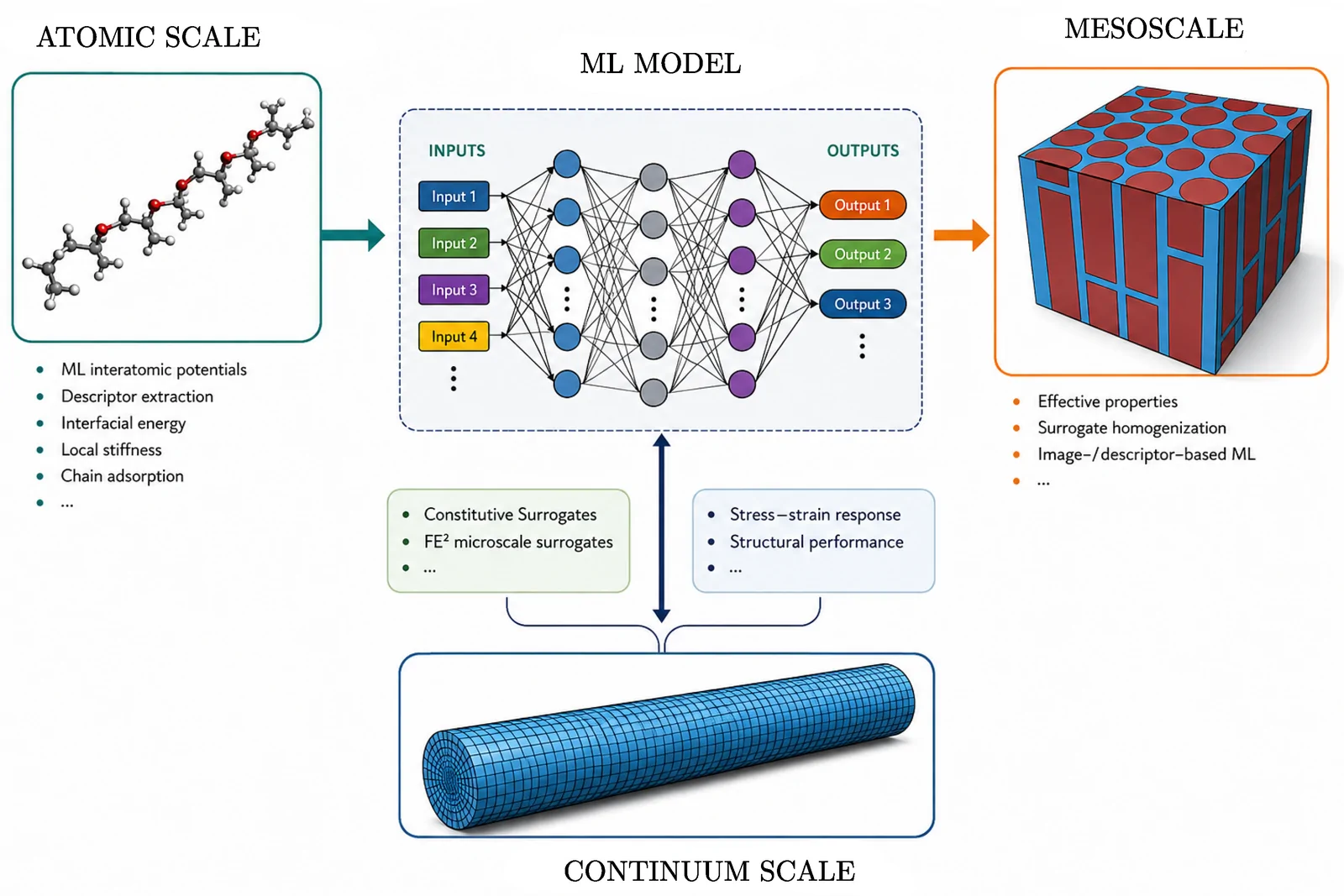
Schematic overview of how ML is integrated across atomistic, mesoscale, and continuum scales for modelling polymer composites and nanocomposites.

**Table 1:** Representative synthetic–synthetic fibre-reinforced polymer hybrid materials and their reported mechanical characteristics.

Hybrid System	Reported Outcome	Mathematical Modelling Relevance	References
Glass–Kevlar–Carbon fibre	Improved flexural strength	Suitable for analytical laminate theories and multiscale finite element modelling because of the different elastic properties of the constituent fibres.	[11,12,13,14]
Kevlar–Glass fibre	Increase in impact strength	Useful for constitutive and progressive damage models describing impact energy absorption and failure mechanisms.	[15,16]
Glass–Carbon fibre	High-modulus carbon layers improve stiffness-to-weight ratio	Frequently employed for micromechanical modelling, rule-of-mixtures validation, and laminate stiffness prediction.	[4,17,18,19,20,21]
Carbon–Kevlar fibre	Improved tensile strength	Suitable for mathematical prediction of tensile behaviour, stress transfer, and failure initiation.	[22]
Reduced Graphene Oxide–Graphene Oxide fibre	Enhanced damping capability	Provides opportunities for multiphysics modelling involving viscoelasticity, damping, and vibration analysis.	[23]

**Table 2:** Representative natural–synthetic fibre-reinforced polymer hybrid materials and their reported mechanical characteristics.

Hybrid System	Reported Outcome	Mathematical Modelling Relevance	Ref.
Glass–Cotton fibre	Increases tensile strength, impact strength and elastic modulus	Suitable for constitutive modelling and elastic parameter identification under tensile and impact loading.	[32]
Glass–Hemp fibre	Toughness increases	Applicable to fracture mechanics and progressive damage modelling.	[38]
Kevlar–Kenaf fibre	Fatigue strength increases	Useful for fatigue life prediction, cyclic damage evolution and durability modelling.	[39,40,41]
Glass–Hemp–Basalt–Flax fibre	Impact strength improved	Suitable for multiscale impact modelling and energy absorption analysis.	[42]
Glass–Kenaf fibre	Improved flexural, tensile and impact strength	Applicable to laminate theory, stiffness prediction and finite element validation.	[30,43,44]
Carbon–Flax fibre	Flexural strength improved	Useful for stiffness prediction, interface modelling and bending analysis.	[28,34]
Glass–Jute fibre	Relatively low elastic modulus	Suitable for homogenization methods and constitutive parameter calibration.	[45,46]
Carbon–Basalt fibre	Improved impact resistance	Applicable to impact mechanics and progressive failure modelling.	[47,48,49]
Kevlar–Basalt fibre	Enhanced energy absorption capability	Useful for crashworthiness simulations and dynamic response modelling.	[50]
Glass–Flax fibre	Improved mechanical properties	Suitable for multiscale constitutive modelling and stiffness evaluation.	[25,51]
Glass–Purpureum Grass fibre	Alkali-treated fibres exhibit higher tensile and flexural strength	Useful for modelling chemically treated natural-fibre composites.	[52]
Glass–Sisal fibre	Improved interfacial bonding and impact strength	Applicable to cohesive-zone modelling and interface damage mechanics.	[53,54]
Glass–Curaua fibre	Hardness and impact properties increase	Suitable for impact response and contact mechanics analysis.	[55]
Glass–Tamarind fibre	Chemical resistance, hardness and impact strength improved after alkali treatment	Useful for durability modelling and coupled chemo-mechanical analyses.	[56]
Honeycomb Core–Carbon fibre	Improved damping performance	Suitable for vibration modelling and sandwich structural analysis.	[57]
Polydopamine–Reduced Graphene Oxide	Improved vibration and damping absorption	Applicable to multiphysics modelling involving viscoelasticity and nanocomposites.	[58,59]
Glass–Bamboo fibre	Storage modulus increases and damping ratio decreases	Suitable for dynamic mechanical analysis and viscoelastic constitutive modelling.	[60]
Glass–Sisal–Jute fibre	Improved tensile and flexural strength	Useful for analytical laminate models and finite element verification.	[61]

**Table 3:** Representative natural–natural fibre-reinforced polymer hybrid materials and their reported mechanical characteristics.

Hybrid System	Reported Outcome	Mathematical Modelling Relevance	References
Banana–Sisal fibre	Improved fibre–matrix interaction	Suitable for constitutive modelling of fibre–matrix interfaces and micromechanical stress-transfer analysis.	[73]
Banana–Kenaf fibre	Mechanical strength increases	Applicable to homogenization methods and finite element prediction of tensile and flexural behaviour.	[64]
Oil Palm–Jute fibre	High flexural and impact strength	Useful for laminate stiffness prediction and impact mechanics modelling.	[74]
Basalt–Flax fibre	Improved durability of natural fibre composites	Suitable for environmental durability modelling and degradation analysis of hybrid composites.	[66,75]
Banana–Coconut fibre	Mechanical properties enhanced	Applicable to stacking-sequence optimization and finite element modelling of laminated composites.	[65]
Kenaf–Bamboo–Coir fibre	Flexural strength and ductility improved	Useful for multiscale constitutive modelling and prediction of mechanical response.	[76]
Jute–Sisal fibre	Storage and loss modulus increase with increasing frequency	Suitable for dynamic mechanical analysis and viscoelastic constitutive modelling.	[77]
Jute–Gongura fibre	Improved tensile strength	Applicable to tensile constitutive modelling and failure prediction.	[78]
Sisal–Cotton fibre	Mechanical and damping properties increase	Useful for vibration modelling and dynamic response analysis of natural fibre composites.	[79]
Banana–Jute fibre	Reduction in energy consumption	Suitable for energy absorption modelling and sustainable lightweight structural design.	[80]
Basalt–Jute fibre	Higher thermal degradation temperature and improved interfacial interaction	Applicable to thermo-mechanical constitutive modelling and interface damage analysis.	[81]

**Table 4:** Comparative mathematical description of polymer hybrid materials according to reinforcement source.

Hybrid System	Mathematical Approaches	Modelling Considerations	References
Synthetic–synthetic	Laminate theory, micromechanics, homogenization, and FEM	Well-characterized constituent properties, fibre fraction, arrangement, stress transfer, and interfacial behaviour.	[2,3,6]
Natural–synthetic	Micromechanics, homogenization, constitutive models, and FEM	Property contrast, natural-fibre variability, morphology, moisture sensitivity, orientation, and interfacial adhesion.	[4,5,6]
Natural–natural	Micromechanics, constitutive models, and experimentally informed multiscale modelling	Variability in both reinforcing phases, morphology, porosity, orientation, processing, and interfacial behaviour.	[4,6]

**Table 5:** Representative mechanical characteristics of polymer hybrid materials reported in the literature.

Polymer Hybrid Material	Reported Outcome	Mathematical Modelling Relevance	References
Jute–Pineapple leaf–Glass fibre	Tensile modulus increases with increasing fibre volume fraction	Suitable for constitutive modelling and prediction of tensile stiffness as a function of fibre volume fraction.	[89]
Sisal/Glass and Sisal/Carbon fibre	Tensile modulus increases with increasing carbon-to-glass fibre ratio	Useful for micromechanical modelling and rule-of-mixtures validation for hybrid laminates.	[90]
Short Coir–Glass fibre	Tensile strength increases with increasing coir fibre content	Applicable to fibre-length-dependent constitutive models and stress-transfer analysis.	[91]
Bamboo–Carbon Nanotube (CNT)	Tensile strength increases with CNT content	Suitable for multiscale modelling of nano-reinforced hybrid composites.	[92]
Banana fibre–Red Mud	Tensile strength increases with increasing red mud content	Useful for particulate-reinforced constitutive modelling and optimization of filler concentration.	[95]
S-Glass–Carbon fibre	Flexural modulus decreases as glass fibre content increases	Applicable to laminate bending theory and stiffness prediction.	[96]
Coir–E-Glass fibre	Impact strength improves through fibre hybridization	Suitable for impact energy absorption and progressive damage modelling.	[91]
Smart Thermoplastic Hybrid Composite (SMA reinforced)	Damage resistance and ductility increase under impact loading	Useful for coupled thermo-mechanical and smart material modelling.	[103]
Kapok–Glass–Sisal fibre	Impact strength increases by approximately 34%	Applicable to impact mechanics and energy absorption prediction.	[107]
Sisal–Coir fibre with *Samanea saman* Pod (SSP)	Flexural and tensile strengths increase with SSP content up to an optimum value	Useful for optimization models considering filler concentration and mechanical response.	[100]
Oil Palm Empty Fruit Bunch (EFB)–Woven Jute	Hybridization improves impact behaviour	Suitable for analytical impact modelling and hybrid laminate optimization.	[101]
E-Glass–PVA fibre	Intraply hybrids provide greater resistance to impact crack propagation	Applicable to crack propagation and fracture mechanics models.	[106]
Flax–Glass and Flax–Carbon fibre	Flax–Glass hybrids exhibit superior impact resistance after impact compared with flax–carbon hybrids	Useful for compression-after-impact modelling and damage tolerance prediction.	[104]

**Table 6:** Fibre properties used in the analytical models.

Fibre	$R_{f}$ (μm)	$E_{f}$ (GPa)	$\sigma_{0}$ (MPa)	*m*	$l_{0}$ (mm)	Reference
AS4 carbon	234	3.5	4275	10.7	12.7	[116]
T300 carbon	232	3.5	3170	5.1	25	[116]
M50S carbon	480	2.65	4600	9	10	[117]
AR glass	70	7	1363	9.6	60	[118]

**Table 7:** Comparative assessment of micromechanical and homogenization models for polymer hybrid materials.

Model and References	Key Assumptions	Applicability	Calibration Requirements	Predictive Limitations
Rule of Mixtures (Voigt) [128,131,132]	Iso-strain; perfect interface; fibres and matrix deform equally	Longitudinal modulus of unidirectional composites	Fibre and matrix moduli; fibre volume fraction	Overestimates transverse and shear moduli
Rule of Mixtures (Reuss) [129,131,132]	Iso-stress; perfect interface; equal stress in constituents	Transverse modulus of unidirectional composites	Fibre and matrix moduli; fibre volume fraction	Underestimates transverse modulus
Hashin–Shtrikman Bounds [134,135,136]	Isotropic constituents; variational principles; polarization tensor	Upper and lower bounds for multiphase composites	Bulk and shear moduli; volume fractions	Bounds wide for high-contrast materials
Chamis Model [137,138,139]	Square fibre packing; square fibre cross-section; RVE subdivision	Transverse and shear modulus prediction	Fibre and matrix moduli; fibre volume fraction	Assumes square fibre cross-section; provides lower bound
Eshelby Inclusion Theory [145]	Ellipsoidal inclusions; elastic matrix; dilute suspension	Stress/strain fields around inclusions; basis for mean-field models	Inclusion geometry; elastic properties	Fails at high volume fractions; neglects inclusion interactions
Mori–Tanaka Model [141,146]	Finite inclusion concentration; average matrix strain as reference	Effective properties with moderate inclusion content	Inclusion geometry; elastic properties; volume fraction	Less accurate for high volume fractions; assumes aligned inclusions
Self-Consistent Scheme [147]	Inclusion embedded in effective medium; no matrix–inclusion distinction	High inclusion content; polycrystalline materials	Elastic properties of phases; volume fraction	May produce non-physical results for porous materials

**Table 8:** Comparative assessment of continuum damage and fracture models for polymer hybrid materials.

Model and References	Key Assumptions	Applicability	Calibration Requirements	Predictive Limitations
Continuum Damage Mechanics (CDM) [154,155,156]	Internal damage variable; thermodynamic framework; isotropic/anisotropic degradation	Distributed damage; progressive degradation; fatigue and creep	Damage evolution law; material parameters; critical damage threshold	Does not explicitly track cracks; mesh-dependent without regularization
Phase-Field Method (PFM) [158,159,160]	Diffuse crack representation; variational energy minimization; regularization length scale	Complex crack patterns; crack branching/merging; heterogeneous materials	Fracture energy; length scale parameter; degradation function	Computationally demanding; length scale sensitivity; parameter calibration challenging
Cohesive Zone Model (CZM) [162,166,167]	Traction–separation law; predefined crack path; damage initiation and evolution	Interfacial delamination; adhesive joints; fibre–matrix debonding	Cohesive strength; fracture energy; penalty stiffness	Requires predefined crack path; cannot predict arbitrary crack initiation

**Table 9:** Comparative assessment of numerical methods for polymer hybrid materials.

Method and References	Key Assumptions	Applicability	Calibration Requirements	Predictive Limitations
Finite Difference Method (FDM) [175,181]	Regular grid discretization; approximates derivatives via finite differences	Simple geometries; layered composites; heat transfer problems	Grid resolution; boundary conditions; material properties	Limited to regular geometries; difficult for complex interfaces and anisotropy
Finite Element Method (FEM) [182,183]	Domain discretization into elements; interpolation functions; variational formulation	Complex geometries; heterogeneous materials; multi-physics problems	Mesh refinement; material properties; boundary/interface conditions	Mesh-dependent results; requires careful validation; high cost for large-scale problems
Boundary Element Method (BEM) [176,186]	Boundary integral formulation; only boundary discretization; fundamental solutions	Stress concentrations; fracture mechanics; infinite/semi-infinite domains	Boundary conditions; fundamental solutions; material properties	Less efficient for non-linear and heterogeneous problems; requires fundamental solutions

**Table 10:** Comparison of principal fracture modelling approaches for polymer hybrid materials.

Method	Formulation	Scale	Fracture Representation	Suitability
XFEM [191]	Enriched FEM approximation with additional functions for displacement discontinuities.	Meso–macro	Explicit crack independent of element boundaries.	Complex crack propagation with unknown crack paths.
CZM [162]	Interface traction–separation law with progressive damage evolution.	Meso–macro	Progressive interfacial decohesion.	Fibre–matrix debonding and interlaminar delamination.
Phase-field [158,159,160]	Variational energy formulation with a regularized continuous damage field.	Meso–macro	Diffuse crack without explicit crack tracking.	Crack branching, merging, and interacting damage in heterogeneous hybrids.
Molecular dynamics [192]	Atomistic equations of motion governed by interatomic interactions.	Atomistic	Bond breaking and atomic rearrangement.	Nanoscale constituent and interfacial mechanisms; limited for structural-scale analysis.

**Table 11:** Comparative overview of theoretical, computational, experimental, and machine-learning approaches for polymer hybrid materials.

Approach and References	Principal Methods	Scale	Main Outputs	Main Limitations
**Theoretical/analytical** [128,129,134,135,136,145,146,147,149]	Rule of mixtures, Halpin–Tsai, Hashin–Shtrikman, Mori–Tanaka, self-consistent and Eshelby models	Micro–macro	Effective stiffness and elastic properties; constituent and volume-fraction effects	Simplifying assumptions regarding microstructure, orientation, and interfaces
**Micromechanical/multiscale** [7,8,120,150,184]	RVE-based modelling, homogenization, micromechanics, and interphase modelling	Micro–meso	Local stress transfer, effective properties, and microstructure–property relationships	Sensitive to morphology, dispersion, interfacial properties, and RVE selection
**Continuum numerical** [173,174,175,176,177,181,182,183]	FDM, FEM, and BEM	Meso–macro	Stress/strain fields, deformation, stiffness, and structural response	Discretization, constitutive assumptions, and computational cost
**Fracture/damage** [154,155,156,158,159,160,162,166,167,191]	CDM, phase-field, CZM, and XFEM	Meso–macro	Damage evolution, crack propagation, delamination, and debonding	Requires fracture parameters and appropriate damage/crack formulation
**Molecular dynamics** [187,188,189,190,192]	Atomistic simulation, molecular interactions, and bond breaking	Atomistic–nano	Interfacial strength, molecular deformation, and fracture-related properties	High computational cost and limited length/time scales
**Experimental** [89,90,91,92,93,96,98,104]	Tensile, flexural, impact, and mechanical characterization	Specimen–macro	Mechanical properties and failure response	Experimental variability, specimen dependence, and testing cost
**Machine learning/data-driven** [126,208,214]	Neural networks, surrogate models, and data-driven prediction	Micro–macro	Property prediction, surrogate models, and accelerated analysis	Requires representative data; limited extrapolation and validation

## Data Availability

No new data were created or analyzed in this study. Data sharing is not applicable to this article.
